# Dynamic Regulation Genes at Microtubule Plus Ends: A Novel Class of Glioma Biomarkers

**DOI:** 10.3390/biology12030488

**Published:** 2023-03-22

**Authors:** Wenwen Wang, Weilong Li, Lifang Pan, Lingjie Li, Yasi Xu, Yuqing Wang, Xiaochen Zhang, Shirong Zhang

**Affiliations:** 1 School of Pharmaceutical Sciences, Zhejiang Chinese Medical University, Affiliated Hangzhou First People’s Hospital, Hangzhou 310053, China; 2 Translational Medicine Research Center, Key Laboratory of Clinical Cancer Pharmacology and Toxicology Research of Zhejiang Province, Affiliated Hangzhou First People’s Hospital, Zhejiang University School of Medicine, Hangzhou 310006, China; 3 The Fourth Clinical Medical College, Zhejiang Chinese Medical University, Affiliated Hangzhou First People’s Hospital, Hangzhou 310006, China; 4 Department of Medical Oncology, The First Affiliated Hospital, College of Medicine, Zhejiang University, Hangzhou 310006, China

**Keywords:** glioma, microtubule plus-end-related gene, biomarker, tumor microenvironment, immunotherapy, drug response

## Abstract

**Simple Summary:**

Microtubule plus-end-related genes (MPERGs) encode a group of proteins that specifically aggregate at the microtubule plus ends to play critical biological roles in the cell cycle, cell movement, ciliogenesis, and neuronal development by coordinating microtubule assembly and dynamics; however, the MPERG correlations and their clinical significance in glioma are not fully understood. This study is the first to systematically analyze and define a seven-gene signature (*CTTNBP2*, *KIF18A*, *NAV1*, *SLAIN2*, *SRCIN1*, *TRIO*, and *TTBK2*) and nomogram model closely associated with clinical factors and the tumor microenvironment as a reliable and independent prognostic biomarker to guide personalized choices of immunotherapy and chemotherapy for glioma patients.

**Abstract:**

Glioma is the most prevalent and aggressive primary nervous system tumor with an unfavorable prognosis. Microtubule plus-end-related genes (MPERGs) play critical biological roles in the cell cycle, cell movement, ciliogenesis, and neuronal development by coordinating microtubule assembly and dynamics. This research seeks to systematically explore the oncological characteristics of these genes in microtubule-enriched glioma, focusing on developing a novel MPERG-based prognostic signature to improve the prognosis and provide more treatment options for glioma patients. First, we thoroughly analyzed and identified 45 differentially expressed MPERGs in glioma. Based on these genes, glioma patients were well distinguished into two subgroups with survival and tumor microenvironment infiltration differences. Next, we further screened the independent prognostic genes (*CTTNBP2*, *KIF18A*, *NAV1*, *SLAIN2*, *SRCIN1*, *TRIO*, and *TTBK2*) using 36 prognostic-related differentially expressed MPERGs to construct a signature with risk stratification and prognostic prediction ability. An increased risk score was related to the malignant progression of glioma. Therefore, we also designed a nomogram model containing clinical factors to facilitate the clinical use of the risk signature. The prediction accuracy of the signature and nomogram model was verified using The Cancer Genome Atlas and Chinese Glioma Genome Atlas datasets. Finally, we examined the connection between the signature and tumor microenvironment. The signature positively correlated with tumor microenvironment infiltration, especially immunoinhibitors and the tumor mutation load, and negatively correlated with microsatellite instability and cancer stemness. More importantly, immune checkpoint blockade treatment and drug sensitivity analyses confirmed that this prognostic signature was helpful in anticipating the effect of immunotherapy and chemotherapy. In conclusion, this research is the first study to define and validate an MPERG-based signature closely associated with the tumor microenvironment as a reliable and independent prognostic biomarker to guide personalized choices of immunotherapy and chemotherapy for glioma patients.

## 1. Introduction

Glioma derived from neuroglial cells and neurons are the most prevalent as well as aggressive primary intracranial nervous system tumors, accounting for 40–50% of intracranial tumors. The annual incidence of glioma in China is 3–6.4 per 100,000 individuals, accounting for 23.3% of central nervous system (CNS) tumors as well as 78.3% of malignant CNS tumors, and the annual mortality rate is about 30,000 individuals [1,2]. According to the 2016 World Health Organization (WHO) classification, glioma can be divided into four different grades based on the malignancy level. Among them, WHO grade I is benign glioma with a long survival period; WHO grades II and III belong to low-grade glioma (LGG) with a median survival of 5–10 years; and WHO grade IV, also known as glioblastoma (GBM), is a high-grade glioma with high invasive and therapeutic resistance and less than two years’ median survival [3]. Genetic and environmental factors are the main risk factors, such as genetic mutation, nitrite-containing food, virus, or bacterial infection, especially high-dose ionizing radiation. Currently, surgical resection plus radiotherapy, chemotherapy, as well as tumor-treating fields are the main treatment methods for glioma. Despite the availability of multiple biomarkers for glioma diagnosis and therapy, the clinical outcome remains poor [4]. Therefore, more sensitive biomarkers and effective therapeutic strategies are critically required to curb glioma progression and improve patients’ outcomes.

The tumor microenvironment (TME) primarily contains peripheral blood vessels, immune and stromal cells, as well as non-cellular substances. At different stages of tumor development, different microenvironment components play a leading role, thus affecting the prognosis and treatment of tumors [5]. For instance, stromal components tend to form a tumor-promoting microenvironment, while immune components are initially recruited by tumor cells and form anti-tumor immune microenvironments. However, as tumors grow, the effector immune cells gradually become exhausted and begin to produce a suppressed immune microenvironment to promote tumor growth and invasion. The blood–brain barrier is initially considered a limiting factor for preventing immune cells from entering the CNS. However, with the discovery of lymphatic components in the CNS, the concept of the CNS as an immune-blind area has gradually weakened, rendering immunotherapy a possible and promising treatment option for glioma [6,7].

A microtubule (MT) is one of the most important cytoskeleton components for maintaining cell morphology, and it is also the main molecular target of tumor drug therapy [8,9,10]. As the core regulator of MT dynamics and the interaction between MT and other organelles or structures, MT plus-end binding proteins (MPEBPs) are a group of proteins that specifically aggregate at the plus end of MTs, and their biological functions in various cell types and stages have been extensively studied [11,12,13]. For instance, in mitotic cells, MPEBPs coordinate mitotic spindle dynamics to ensure bipolar orientation and the precise separation of chromosomes, which is critical for maintaining genomic stability. In non-mitotic cells, MPEBPs are mainly responsible for regulating cell polarization, migration, communication, and movement to maintain the normal structure of cells, contributing to the normal occurrence of various physiological processes, including ciliogenesis and neuronal development. Mutation or abnormal regulation of MT plus-end-related genes (MPERGs) leads to a series of MT-related diseases, including ciliopathies and neurodevelopmental and neurodegenerative diseases [12,14]. At present, a growing number of MPERGs have been found to be expressed abnormally in malignant tumors, thus affecting the growth, migration, and invasion of tumors. The dynamic change in the MT plus end also determines the sensitivity of MT-targeted drugs, resulting in different drug responses in patients [15]. Although it is relatively convenient to analyze the function of individual MPERGs in tumors, the systematic study of the multi-gene signature in glioma could better reveal the impact of their overall relationship on glioma. Additionally, targeted treatment for individual MPERGs is usually insufficient to achieve the expected clinical results. Therefore, the comprehensive study of MPERGs will guide multi-target combined therapy, thus benefiting more glioma patients.

**Figure 1:**
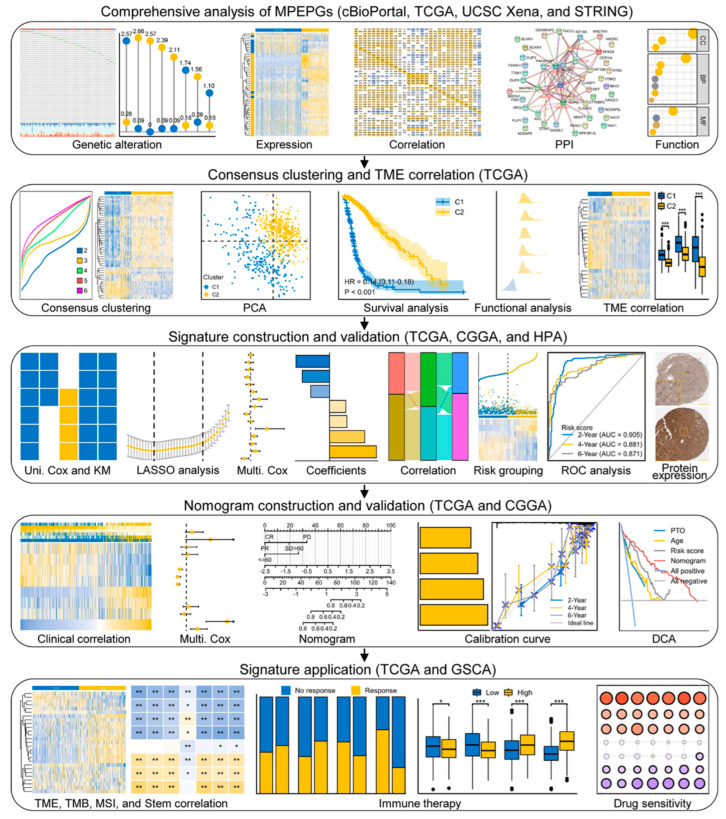
Flowchart of the research.

In this study, we used public datasets to systematically analyze MPERG expression, focusing on their clinical significance in glioma. We aimed to develop a novel MPERG-based prognostic signature and evaluate its potential to anticipate the clinical outcome and therapeutic effect in glioma patients. Figure 1 presents a flowchart of the research.

## 2. Materials and Methods

### 2.1. Data Acquisition

The mRNA sequencing and matched clinical data of normal and glioma (LGG and GBM) specimens were gathered from The Cancer Genome Atlas (TCGA, https://portal.gdc.cancer.gov/, accessed on 20 June 2022) (5 para-tumors and 698 glioma tissues), University of California Santa Cruz (UCSC) Xena (https://xenabrowser.net, accessed on 20 June 2022) (1152 normal, 5 para-tumors, and 689 glioma tissues), and Chinese Glioma Genome Atlas (CGGA, http://www.cgga.org.cn/, accessed on 7 October 2022) (325 and 693 cases of mixed glioma tissues) databases, respectively. Samples lacking clinical information or duplicated testing were excluded [16,17,18]. The R program (v 3.6.3) and ggplot2 R tool (v 3.3.3) were utilized for the data analysis and observation, respectively.

### 2.2. Genetic Alteration and Methylation Analyses

The oncoprint of somatic mutation, histograms and Kaplan–Meier (KM) plots of genetic alteration were directly extracted from cBioPortal (https://www.cbioportal.org/, accessed on 20 June 2022) (TCGA, Firehose). The copy number alteration (CNA) frequencies and Spearman correlation between the MPERG expression and CNA and DNA methylation were also recorded from cBioPortal and visualized using lollipop and heatmap, respectively [19,20,21].

### 2.3. Differential Expression Analyses

The distribution of the gene expression was displayed by complex heatmaps using the ComplexHeatmap R package (v 2.2.0) [22]. The comparison of the gene expression in different tissues or groups was displayed using histograms through the Wilcoxon rank-sum test. Immunohistochemical (IHC) images of the signature protein expression in healthy and glioma samples were extracted from the Human Protein Atlas (HPA) portal (https://www.protein.org, accessed on 20 June 2022).

### 2.4. Protein–Protein Interaction Analyses

The STRING portal (https://www.string-db.org/, accessed on 20 June 2022) was utilized to get the protein–protein interaction (PPI) network with high confidence (minimum interaction score = 0.7) [23].

### 2.5. Unsupervised Consensus Clustering

Unsupervised consensus clustering of the glioma patients was carried out using the ConsensusClusterPlus R package (v 1.54.0) with 1000 repetitions, and the cumulative distribution function (CDF) curve was employed to gain the appropriate cluster number. A principal component analysis (PCA) was employed to display the distribution differences among the clusters. A KM plot indicating the survival difference was obtained with the survivin R package (v 3.2-10) and displayed with the survminer R package (v 0.4.9) [24,25,26].

### 2.6. Functional Enrichment Analyses

First of all, the MPERGs were directly used for the Gene Ontology (GO) and Kyoto Encyclopedia of Genes and Genomes (KEGG) functional studies with the clusterProfiler R package (v 3.14.3). The cluster-associated genes were identified with the limma R package (v 3.40.2) and visualized using a volcano plot [27]. Then, the cluster-associated genes were further used for a Gene Set Enrichment Analysis (GSEA) containing the h.all.v7.2 (Hallmark) and c2.cp.v7.2 (KEGG) gene sets with the clusterProfiler R package [28,29].

### 2.7. Development and Validation of Risk Signature and Nomogram Model

The independent prognostic genes for constructing the risk signature were screened from 45 DEMPERGs using the univariate, KM, least absolute shrinkage and selection operator (LASSO), and multivariate Cox regression methods in turn with the survival and glmnet R (v 4.1-2) packages [17]. The histograms representing the risk coefficient and concordance index (C-index) were obtained from the Cox regression analyses. The receiver operating characteristic (ROC) curves representing the prognostic value were generated with the pROC (v 1.17.0.1) and timeROC R (v 0.4) packages, while the risk score plot was generated with the ggrisk R package (v 1.3). The correlation among the clusters, risk scores, and survival statuses was displayed by means of a Sankey plot with the ggalluvial R package (v 0.12.3).

The distributions of the risk signature gene expressions and risk scores in diverse clinical statuses were visualized using complex heatmaps. The risk grouping under different clinical factors was compared utilizing a chi-squared test and displayed using a baseline datasheet. To finally screen the independent prognostic factors, uni- and multi-variate Cox regression analyses were carried out and displayed by means of a forest plot. The nomogram model was then developed based on these factors using the rms (v 6.2-0) and survival R packages. Calibration curves and the C-index were used to assess the accuracy of the prediction. The decision curve analysis (DCA) plot for assessing the clinical net benefit was generated with the survival R package and stdca R [30].

### 2.8. TME Infiltration Analyses

The TME infiltration, including the stromal and immune cell infiltration, was calculated via the ssGSEA method in the GSVA package (v 1.34.0), the Estimate package (v 1.0.13), and the xCell method in the immunedeconv package, and visualized using complex heatmaps [31,32,33,34]. The infiltrations in various cells or groups were compared utilizing the Wilcoxon rank-sum test and displayed via histogram. The Spearman correlations between the signature as well as the TME infiltration, tumor mutation burden (TMB), and microsatellite instability (MSI) were examined and visualized using heatmaps [35,36,37]. A stemness analysis was conducted using the OCLR algorithm, and the Spearman correlations between the signature and stemness were visualized using heatmaps [38].

### 2.9. Immunotherapy and Drug Sensitivity Analyses

The Tumor Immune Dysfunction and Exclusion (TIDE, https://tide.dfci.harvard.edu/, accessed on 20 June 2022) portal uses immune escape and immune cell dysfunction to anticipate the immune checkpoint blockade (ICB) treatment response of patients. The TIDE scores in different groups were compared utilizing Welch’s *t*-test and displayed via histograms. High scores indicated stronger immune escape, resulting in poorer ICB efficacy and shorter survival, and vice versa [39,40].

The Gene Set Cancer Analysis (GSCA, http://bioinfo.life.hust.edu.cn/GSCA/#/, accessed on 20 June 2022) portal containing Cancer Therapeutics Response Portal (CTRP) data was employed to anticipate the drug sensitivity according to the connection between the gene expression and half maximal inhibitory concentration (IC50) of small molecules [41]. The correlation heatmap was downloaded from the portal. Positive correlation revealed that increased gene expression was associated with the high IC50 of the drugs, indicating low drug sensitivity and poor therapeutic effect, and vice versa.

## 3. Results

### 3.1. Systematic Analyses of MPERG Expression, Genetic Alteration, Correlation, and Interaction in Glioma

We identified 46 MPERGs, including *AMER2*, *APC*, *APC2*, *CDK5RAP2*, *CEP104*, *CKAP5*, *CLASP1*, *CLASP2*, *CLIP1*, *CLIP2*, *CLIP3*, *CLIP4*, *CTTNBP2*, *DCTN1*, *DST*, *FBXW11*, *FILIP1*, *GAS2L1*, *GAS2L2*, *KIF11*, *KIF18A*, *KIF18B*, *KIF2C*, *KNSTRN*, *MACF1*, *MAPRE1*, *MAPRE2*, *MAPRE3*, *NAV1*, *NAV2*, *NAV3*, *NCKAP5*, *NCKAP5L*, *PAFAH1B1*, *PPP1R13L*, *PSRC1*, *SLAIN1*, *SLAIN2*, *SPAG5*, *SRCIN1*, *STIM1*, *SYBU*, *TACC3*, *TRIO*, *TTBK1*, and *TTBK2*, from the Molecular Signatures Database (MsigDB, https://www.gsea-msigdb.org/gsea/msigdb/index.jsp, accessed on 20 June 2022) with the standard name GOCC_MICROTUBULE_PLUS_END and prior investigations [11,12,13,42,43]. Table 1 presents the gene and protein names and MT plus end binding modes of the MPERGs.

**Figure 2:**
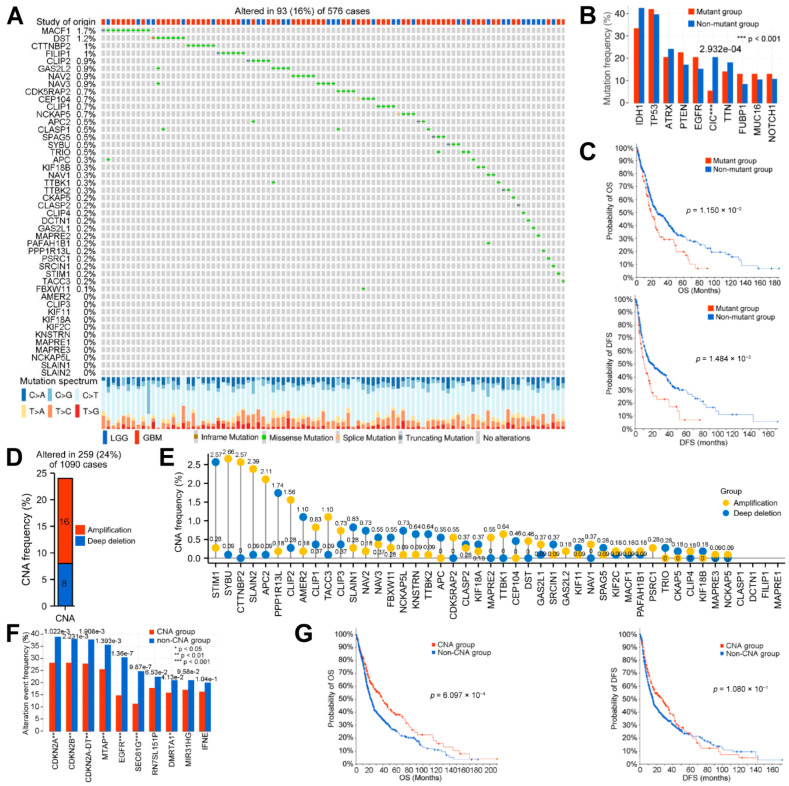
Genetic alteration of the MPERGs in glioma from cBioPortal. (**A**) The oncoprint of the genetic mutations. (**B**,**F**) Histograms of the mutations (**B**) and can (**F**) of other genes between the two groups. (**C**,**G**) KM OS and DFS plots between the two groups. (**D**) Histogram of the total CNA frequency of the MPERGs. (**E**) Lollipop plot of the CNA frequency in the MPERGs.

To study the correlation and function of these genes in glioma, we systematically analyzed them from the aspects of genetic alteration, DNA methylation, mRNA expression, mRNA correlation, protein–protein interaction, and functional correlation. Firstly, we visualized the genetic alteration of the MPERGs, including the single nucleotide alteration (SNA) and can, from cBioPortal. Only 16% (93/576) of patients had MPERG mutations, and the mutation frequency of individual genes was very low (≤1.7%). The mutation styles mainly included in-frame, missense, splice, and truncating mutations (Figure 2A). To further observe the mutations of other genes, we divided the sample into the MPERG mutant group and the non-mutant group. As shown in the histogram, *IDH1*, *TP53*, *ATRX*, *PTEN*, *EGFR*, *CIC*, *TTN*, *FUBP1*, *MUC16*, and *NOTCH* were the ten genes with the highest mutation frequency in any group (Figure 2B; Table S1). Among them, *TP53*, *ATRX*, *PTEN*, *EGFR*, and *NOTCH1* are the 50 most commonly mutated genes in cancer, while *IDH1* mutation is a mark event for the early development of glioma, indicating a favorable prognosis [44,45]. However, only the frequency of the *CIC* mutation had a significant difference between the two groups (*p* = 2.932 × 10^−4^). In addition, the overall survival (OS, *p* = 1.150 × 10^−2^) and disease-free survival (DFS, 1.484 × 10^−3^) in the mutant group were shorter than those in the non-mutant group (Figure 2C). Next, we examined the CNA of the MPERGs. Here, 24% (259/1090) of the patients had MPERG CNAs, and amplification (16%) was more likely to occur than deep deletion (8%) (Figure 2D). Similarly, the CNA frequency of each gene was very low (<3%) (Figure 2E; Table S2). Among these patients, *CDKN2A*, *CDKN2B*, *CDKN2A-DT*, *MTAP*, *EGFR*, *SEC61G*, *RN7SL151P*, *DMRTA1*, *M1R31HG*, and *IFNE* were the ten genes with the highest CNA frequencies, and the frequencies of *CDKN2A*, *CDKN2B*, *CDKN2A-DT*, *MTAP*, *EGFR*, *SEC61G*, and *DMRTA1* were lower in the MPERG CNA group than in the non-CNA group (Figure 2F). Additionally, compared with the non-CNA group, the MPERG CNA group had higher OS (*p* = 6.097 × 10^−4^), while no significant variation in the DFS was detected (Figure 2G). According to our findings, although the genetic alteration of individual MPERGs is very low, the overall alteration of the MPERGs still causes dysfunction, leading to significant prognostic differences in glioma patients.

**Figure 3:**
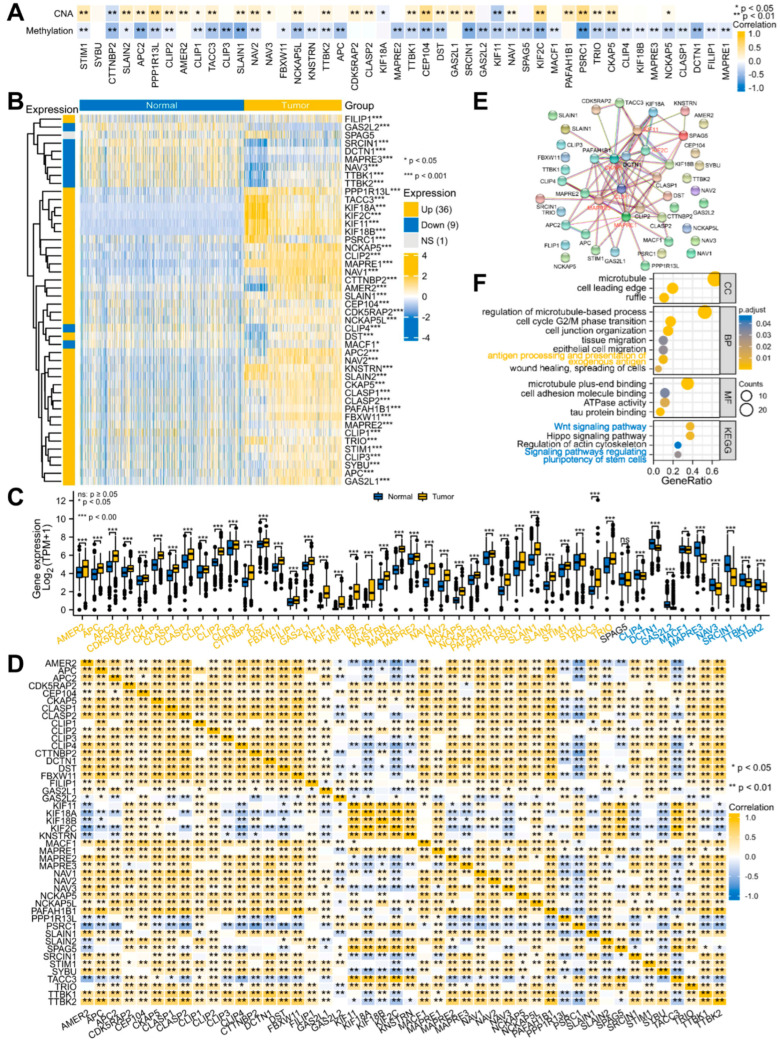
The expression, correlation, interaction, and function of the MPERGs in glioma. (**A**) Heatmap of the Spearman relationship between the MPERG expression and CNA, DNA methylation from cBioPortal. (**B**,**C**) Complex heatmap and histogram of the MPERG expression in normal and glioma tissues from the UCSC Xena database. (**D**) Correlation heatmap of the MPERGs from the TCGA database. (**E**) Interaction network of the MPERG proteins from the STRING database. Red words indicate hub genes. (**F**) Bubble plot of the GO and KEGG analyses. Yellow and blue words represent immune− and cancer−stemness−associated pathways, respectively.

Previous studies have demonstrated that CNA and DNA methylation are crucial factors in regulating gene expression [46,47]. Our correlation results also showed that most MPERGs positively correlated with the CNAs, while they negatively correlated with DNA methylation (Figure 3A; Table S3). Therefore, we further evaluated the MPERG expression between the normal and glioma tissues with TCGA and GTEx data from the UCSC Xena database. Based on the Wilcoxon rank-sum test, 45 differentially expressed MPERGs (DEMPERGs) were found, including 36 upregulated and 9 downregulated genes (Figure 3B,C). Most MPERGs had correlation with each other in glioma (Figure 3D; Table S4). Additionally, the PPI analysis identified MAPRE1, CKAP5, MAPRE3, CLIP1, KIF11, and KIF2C as hub proteins of interaction (node degree ≥ 10) (Figure 3E; Table S5).

To further explore the effect of their correlation and interaction on glioma, we conducted a functional enrichment analysis. The GO functional annotations indicated that the MPERGs were primarily located at the MT, cell leading edge, and ruffle, and they were significantly enriched in the cell cycle, epithelial cell migration (ECM), tissue migration, wound healing, and antigen processing presentation of exogenous antigen. Additionally, the KEGG enrichment analysis indicated that they were enhanced in the Hippo signaling pathway, regulation of actin cytoskeleton, Wnt signaling pathway, and signaling pathways regulating the pluripotency of stem cells. It is noteworthy that the last two pathways are also cancer-stemness-related pathways, implying that the MPERGs may participate in cancer- and immune-related pathways, especially cancer-stemness-related pathways (Figure 3F; Table S6).

### 3.2. Glioma Patients with DEMPERGs Were Well Distinguished into Two Subgroups with Survival and TME Infiltration Differences

**Figure 4:**
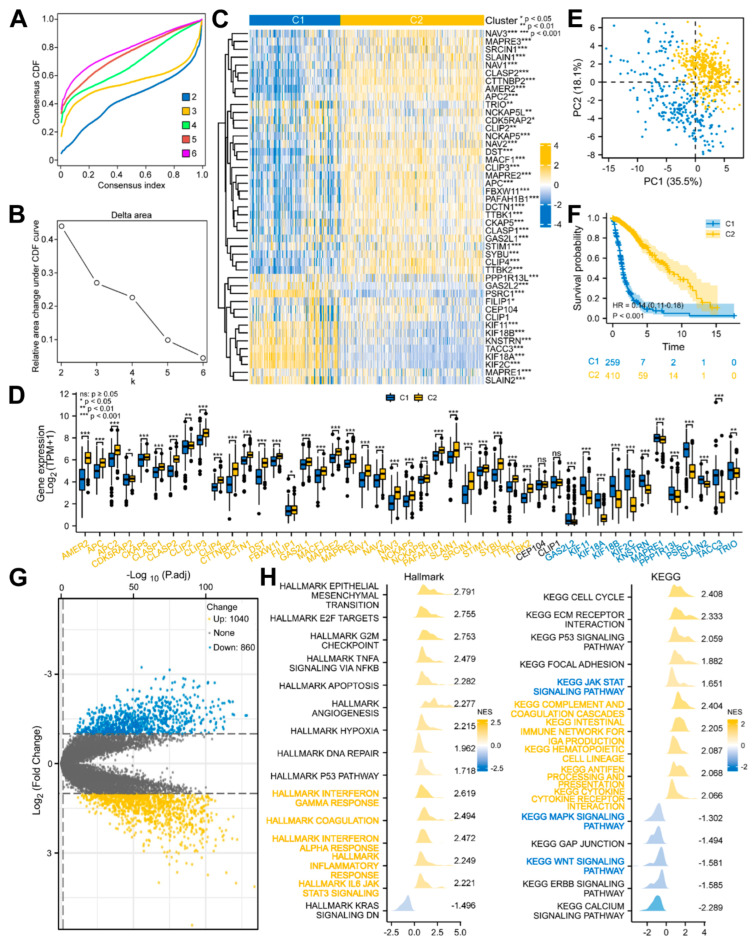
Glioma patients with DEMPERGs were distinguished into two subgroups via unsupervised consensus clustering with differences in survival and function. (**A**) Consensus CDF curve of two to six clusters. (**B**) Relative area changes under the CDF curve from k = 2 to 6. (**C**) Complex heatmap of the DEMPERG expression in two clusters. (**D**) DEMPERG expression between the two clusters. (**E**) PCA plot. (**F**) KM OS plot. (**G**) Volcano plot of the cluster-associated genes. (**H**) Ridge plots of the GSEA. Yellow and blue words represent immune− and cancer−stemness−associated pathways, respectively.

Based on the above-mentioned 45 DEMPERGs, the glioma patients were distinguished into two to six subgroups, where k = 3 was the ideal clustering with the most stable k value (Figure 4A,B and Figure S1A,B). Nevertheless, non-significant variation existed in the OS between clusters 1 and 2 (Figure S1C). Therefore, we re-clustered the patients according to k = 2 for the subsequent analyses (Figure 4C). Forty-three of the 45 genes were expressed differently in two clusters (Figure 4D). The PCA plot confirmed that these two clusters were well distinguished (Figure 4E), and worse OS in cluster 1 was observed than in cluster 2 (Figure 4F).

Subsequently, functional enrichment with GSEA was conducted to evaluate the possible mechanism of survival differences. The result showed that 1040 upregulated and 860 downregulated genes were detected between clusters 1 and 2 (|log_2_fold change| > 1, adjusted *p* < 0.05) (Figure 4G). The cluster-associated genes were positively correlated with the cancer-related hallmarks (e.g., epithelial-mesenchymal transition, G2/M checkpoint, apoptosis, angiogenesis, and p53 pathway) and immune-related hallmarks (e.g., IL6-JAK/STAT3 signaling, interferon response, coagulation, and inflammatory response). From the KEGG, they were strongly related to the cancer-related pathways (e.g., cell cycle, ECM receptor interaction, and p53 and JAT-STAT signaling pathways) and immune-related processes (e.g., antigen processing and presentation, and cytokine–cytokine receptor interaction), and negatively related to the MAPK, Wnt, and ERBB signaling pathways. Among them, the JAK-STAT, MAPK, and Wnt signaling pathways are also cancer-stemness-related pathways (Figure 4H; Table S7). These findings indicated that besides the cell cycle and cell migration, the MPERG-associated genes also participate in the cancer- and immune-associated pathways.

**Figure 5:**
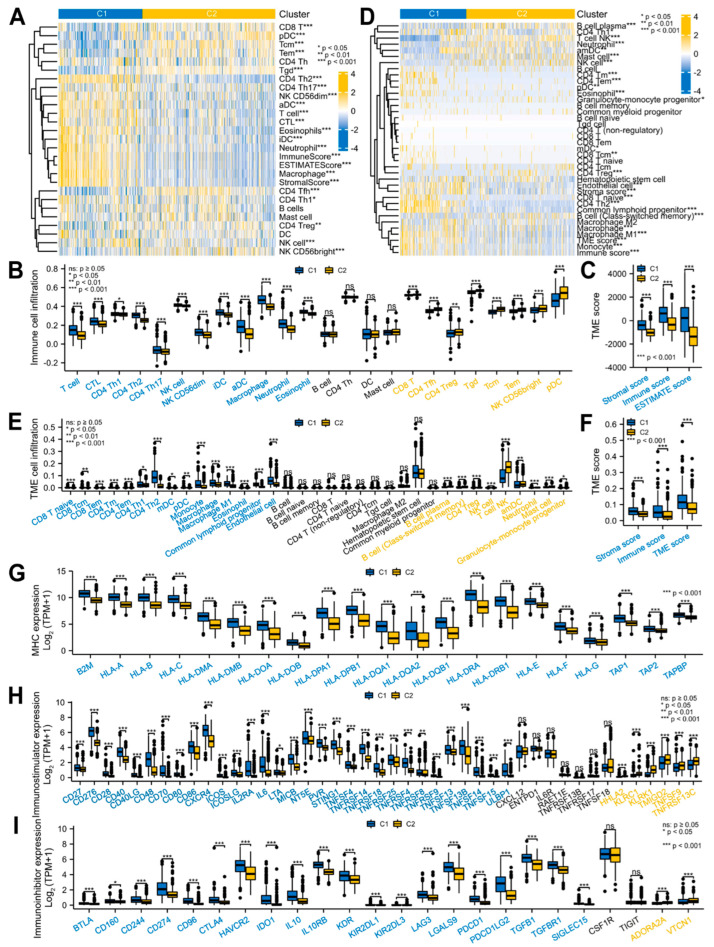
Cluster−associated glioma patients were infiltrated in different TMEs. (**A**–**F**) Complex heatmaps and histograms of the TME infiltration between the two clusters using the ssGSEA and ESTIMATE (**A**–**C**) and xCell (**D**–**F**) algorithms. (**G**–**I**) Histograms of the MHC (**G**), immunostimulator (**H**), and immunoinhibitor (**I**) expression between the two clusters. MHC, major histocompatibility complex.

Considering the close relationship between the MPERG-associated genes and above-mentioned pathways, we investigated the TME infiltration between the two clusters. Based on the ssGSEA method, the T, cytotoxic T lymphocyte (CTL), CD4 T helper (Th) 1/2/17, natural killer (NK), NK CD56 dim cells, immature dendritic cells (iDCs), activated DCs (aDCs), macrophages, neutrophils, and eosinophils were strongly infiltrated in the cluster 1 patients. In contrast, the cluster 2 patients were mainly infiltrated in the CD8 T, CD4 follicle helper T (Tfh), CD4 regulatory T (Treg), gamma delta T (Tgd), central memory T (Tcm), effector memory T (Tem), NK CD56 bright cells, and plasmacytoid DCs (pDCs) (Figure 5A,B). In general, the cluster 1 patients had markedly higher TME scores than the cluster 2 patients, including the stromal, immune, and ESTIMATE scores, as evaluated using the ESTIMATE method (Figure 5A,C). Next, we used the xCell algorithm to verify this result. The patients in cluster 1 showed high infiltrations in the CD8 T naive/Tcm/Tem, CD4 Tm/Tem/Th1/Th2 cells, myeloid DC (mDC), pDC, monocyte, macrophage, macrophage M1, eosinophil, common lymphoid progenitor, and endothelial cells. The cluster 2 patients had high infiltrations in the B cell plasma, B cell (class-switched memory), CD4 Treg, NK, mast cells, T cell NK, activated myeloid DCs (amDCs), neutrophils, and granulocyte-monocyte progenitor cells (Figure 5D,E). The TME scores were the same as the ESTIMATE algorithm scores (Figure 5D,F). Next, we investigated the immune-related gene expression in the two clusters. The expression of the major histocompatibility complexes and most immunostimulators in cluster 1 was higher than that in cluster 2. Surprisingly, most of the immunoinhibitor expression in cluster 1 was also higher, implying that there is a relatively complex immune microenvironment in MPERG-related glioma, and their relationship with prognosis and treatment needs further analysis (Figure 5G–I). In conclusion, the TME infiltration was significantly different between the two subgroups of glioma patients.

### 3.3. A Seven-Gene Prognostic Signature Was Constructed and Validated in Glioma

**Figure 6:**
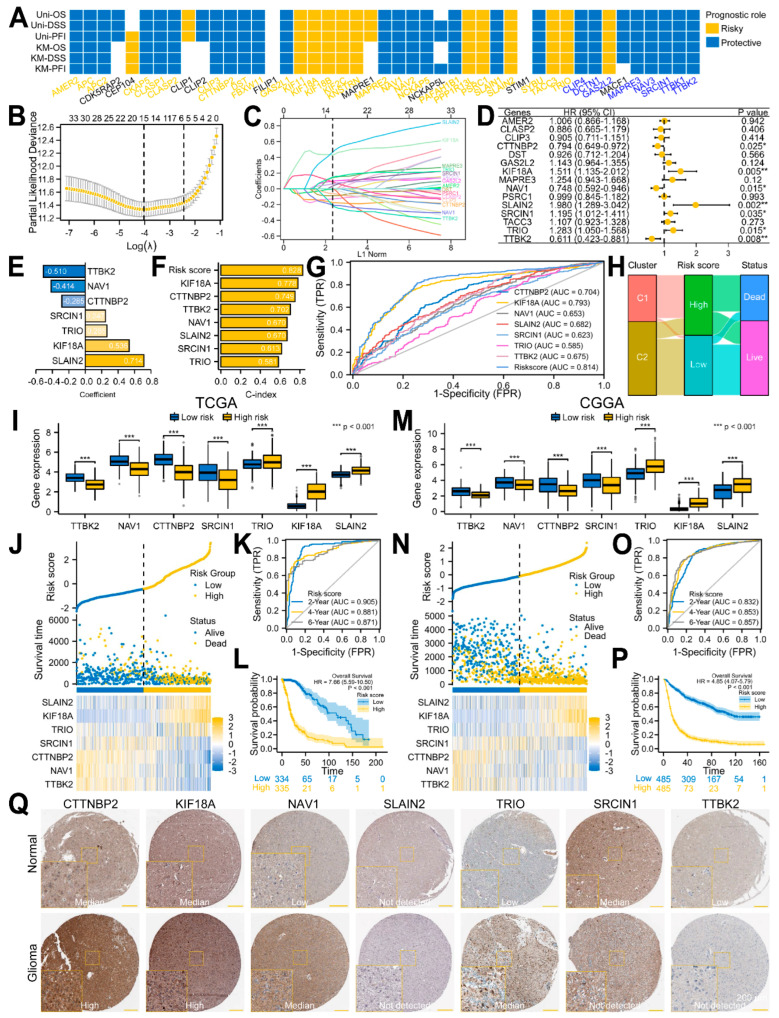
Development and validation of an MPERG−associated prognostic signature in glioma using the TCGA and CGGA datasets. (**A**) Univariate Cox and KM analyses of the DEMPERGs. (**B**) LASSO Cox regression analysis of the prognostic−related DEMPERGs using a ten−fold cross−validation method. (**C**) The solution path plot of the variable against the L1 norm. (**D**) Forest plot of the multivariate Cox regression analysis of the screened genes with OS. * *p* < 0.05, ** *p* < 0.01. (**E**) Bar charts of the variables and their coefficients. (**F**) C-indexes of the regression model. (**G**) ROC analysis of the survival status for the risk signature and associated gene. (**H**) Sankey plot of the correlation among the clusters, risk scores, and survival statuses. (**I**,**M**) Signature gene expression between the two risk groups. (**J**,**N**) Risk score plot. (**K**,**O**) Time−dependent ROC curve. (**L**,**P**) KM OS plot of the two groups. (**Q**) Signature protein expression between the normal and glioma samples.

To screen the predictive factors from the 45 DEMPERGs, we first conducted univariate and KM Cox regression analyses. A total of 11 risk genes and 25 protective genes were identified as having predictive value for the OS, disease-specific survival, as well as progression-free interval (Figure 6A and Figure S2). Therefore, a LASSO Cox regression analysis was performed on these 36 genes. A total of 15 genes were selected after taking the variables with minimized lambda (Figure 6B,C). The multivariate Cox regression analysis of these 15 genes indicated that 7 of them were independent prognostic genes (Figure 6D). Thus, we established a prognostic signature based on these seven gene. $\mathrm{Risk}\;\mathrm{score}\;=\left(-0.510\right)\times TTBK2+\left(-0.414\right)\times NAV1+\left(-0.285\right)\times CTTNBP2+0.247\times SRCIN1+0.265\times TRIO+0.536\times KIF18A+0.714\times SLAIN2\;$(Figure 6E). *TTBK2*, *NAV1*, and *CTTNBP2* were considered as protective factors, while *SRCIN1*, *TRIO*, *KIF18A*, and *SLAIN2* were risk factors for glioma. Compared with a single gene, the seven-gene signature had the highest C-index in the Cox regression analyses (0.828) and area under the curve (AUC) (0.814) in the ROC curve, indicating that it is the most appropriate factor for predicting glioma patients’ clinical outcome (Figure 6F,G). The Sankey plot showed that the patients in cluster 1 had high risk scores and dead status, while the patients in cluster 2 had the opposite (Figure 6H).

We further used the TCGA and CGGA datasets as training and validation sets to assess and validate the risk signature. All the signature genes were expressed differentially between the two groups (Figure 6I,M). Moreover, with the risk score elevated, the survival time became shorter as well as closer to death (Figure 6J,N). The time-dependent ROC analysis revealed that the 2-, 4-, and 6-year OS AUCs in the TCGA were 0.905, 0.881, and 0.871, while the AUCs in the CGGA were 0.832, 0.853, and 0.857, respectively (Figure 6K,O). The KM plot confirmed that the high-risk patients showed a worse OS in comparison with the low-risk patients (Figure 6L,P). In addition, we further verified the signature protein expression in the normal and glioma samples using the HPA database. Compared with the normal samples, except for SLAIN2, the CTTNBP2, KIF18A, NAV1, and TRIO protein expression in glioma was elevated, while SRCIN1 and TTBK2 were decreased (Figure 6Q). Referring to Figure 3B, the signature gene was basically consistent with their protein expression. These findings confirmed that the risk signature we constructed has very high prediction accuracy.

### 3.4. A Predictive Nomogram Model Was Established and Verified to Facilitate the Clinical Application of the Risk Signature in Glioma

**Figure 7:**
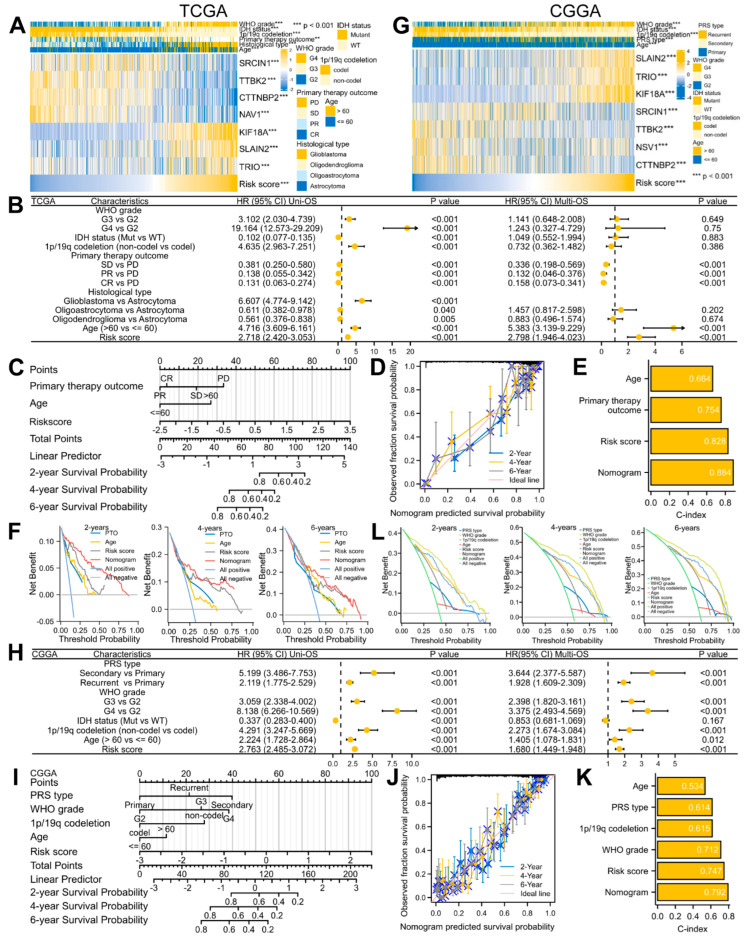
Building and verification of a predictive nomogram model in glioma using the TCGA and CGGA datasets. (**A**,**G**) Complex heatmaps of the risk signature and clinical features. ** *p* < 0.01, *** *p* < 0.001. (**B**,**H**) Forest plots of the uni− and multi−variate Cox regression analyses. (**C**,**I**) Nomogram model of the independent prognostic factors. (**D**,**J**) Calibration analyses of the OS nomogram. (**E**,**K**) C-index plots of the OS nomogram. (**F**,**L**) DCA plots of the OS nomogram.

Due to the strong correlation between the risk score and prognosis, we also incorporated clinical factors into the prognosis model. According to the TCGA dataset, an increased risk score was highly related to the malignant progression of glioma, including *IDH*-WT status, 1p/19q non-codeletion, older age, higher WHO grade, more inclined to form glioblastoma, and worse response to treatment (Table 2; Figure 7A). A similar result was also obtained from the CGGA datasets (Table 3; Figure 7G). To determine whether the signature and clinical features are independent prognostic factors in glioma, we conducted Cox regression analyses and observed that the risk score, PTO, as well as age had independent prognostic value in the TCGA dataset (Figure 7B), while the CGGA datasets presented more factors, including the WHO grade, 1p/19q codeletion, PRS type, age, and risk score (Figure 7H). Therefore, based on these independent prognostic factors, we finally established a nomograph model for predicting the 2-, 4-, and 6-year OS (Figure 7C,I). The results showed that the calibration curve matched the ideal diagonal line well (Figure 7D,J), and the C-index of the nomogram was also better than a single clinical factor or risk signature (0.884 for TCGA; 0.792 for CGGA) (Figure 7E,K). In addition, the DCA showed that the nomogram was the best prognostic indicator (Figure 7F,L). These results demonstrated that our constructed and validated nomogram including clinical factors is the best predictor of glioma prognosis.

### 3.5. Signature-Associated Glioma Patients Were Predicted to Benefit from Immunotherapy and Chemotherapy

**Figure 8:**
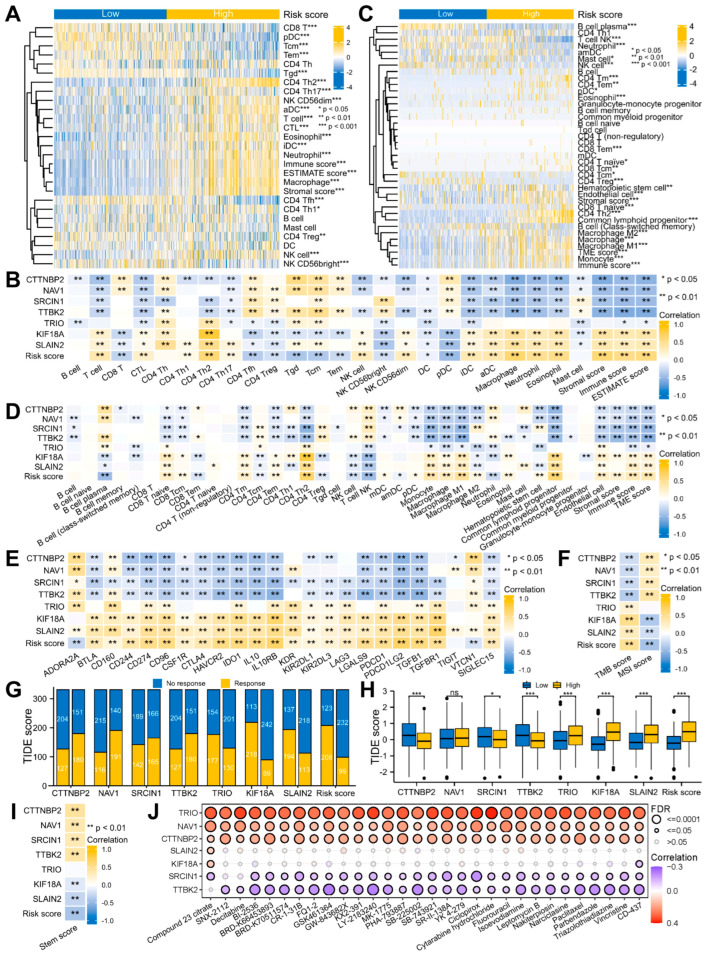
Application of the risk signature to immunotherapy and chemotherapy. (**A**,**C**) Complex heatmaps of the TME infiltration in the two risk groups using the ssGSEA, ESTIMATE (**A**), and xCell (**C**) algorithms. (**B**,**D**–**F**) Heatmaps of the correlation between the risk signature and TME infiltration (**B**,**D**), immunoinhibitors (**E**), TMB and MSI scores (**F**). (**G**,**H**) Histograms of the ICB response (**G**) and TIDE score (**H**) prediction using the TIDE algorithm. ns *p* ≥ 0.05, * *p* < 0.05, ** *p* < 0.01, *** *p* < 0.001. (**I**) Heatmap of the correlation between the risk signature and stem score. (**J**) Heatmap of the correlation between the signature gene and IC50 of drug from the GSCA portal.

Moreover, we continued our investigation into the connection between the risk signature and TME. The ssGSEA method revealed that the infiltrations of the T, CTL, CD4 Th1/2/17, NK, and NK CD56 dim cells, iDCs, aDCs, macrophages, neutrophils, and eosinophils were strongly connected with the risk scores, whereas the CD8 T, CD4 Tfh, CD4 Treg, Tgd, Tcm, Tem, and NK CD56 bright cells, DC and pDC were inversely correlated with them. Overall, the TME scores evaluated using the ESTIMATE algorithm were positively related to the risk scores (Figure 8A,B and Figure S3A,B; Table S8). In addition, similar results were obtained using the xCell algorithm (Figure 8C,D and Figure S3C,D; Table S9).

Subsequently, we continued to explore the response of the glioma patients to immunotherapy and chemotherapy. Immunotherapy is known to be influenced by immunoinhibitor expressions, TMB and MSI scores and other factors [36,37]. Among the 24 immunoinhibitors, 21 of them were strongly related to the risk score (Figure 8E and Figure S3E; Table S10). High TMB and MSI scores indicate that tumor cells have higher antigenicity and more neoantigens, thus having stronger immune infiltration. The outcomes indicated a positive correlation between the risk score and TMB as well as a negative relationship to the MSI (Figure 8F and Figure S3F; Table S11). These results strongly suggested that immunotherapy is effective for glioma. Therefore, TIDE was utilized to anticipate the efficacy of ICB therapy. The result showed that the TIDE score was lesser in the low- compared to the high-risk patients, indicating a better immunotherapy response and treatment outcome for the low-risk patients (Figure 8G,H).

Cancer stem cells are mainly accountable for the maintenance and growth of cancer cells, which are highly correlated with tumor metastasis, recurrence, as well as therapy resistance [48]. The functional enrichment analysis suggested that the MPERGs and MEPRG-associated genes may be involved in cancer stemness. Therefore, we introduced the OCLR algorithm to assess the variations of cancer stemness between both groups. The results presented a negative relationship between the risk and stem score, indicating that the low-risk patients had stronger stem cell characteristics and differentiation potential than the high-risk patients (Figure 8I).

Gene expression may affect the clinical response of patients to drug therapy; hence, these genes represent possible drug screening targets. We studied the connection between the signature expression and drug sensitivity utilizing the CTRP dataset from the GSCA site. The findings revealed that *TTBK2* and *SRCIN* were inversely associated with the IC50 of the majority of the drugs, while *TRIO*, *NAV1*, and *CTTNBP2* positively correlated with them, implying that *TTBK2* and *SRCIN* increased expression or *TRIO*, *NAV1*, and *CTTNBP2* reduced expression could enhance the sensitivity of patients to these drugs (Figure 8J). Therefore, these genes could be new chemotherapeutic targets.

## 4. Discussion

Despite treatment advances, glioma remains a fatal disease. Therefore, new therapeutic targets and methods are urgently needed. In recent years, more and more MPERGs have been found to be aberrantly expressed in several cancers, which could be connected to tumor occurrence, development, and prognosis. In our research, we first thoroughly analyzed 46 MPERGs regulated by CNA and DNA methylation and found that 45 MPERGs were differentially expressed in MT highly enriched glioma. Glioma patients with 45 DEMPERGs could be distinguishably divided into two subgroups with significant prognostic and functional differences. Next, we used univariate, KM, LASSO, and multivariate Cox regression analyses in turn to finally screen seven independent prognostic genes and then constructed the prognostic signature based on the MPERGs for the first time.

The biological functions of these seven signature genes have been extensively studied, although their correlation with cancer, especially glioma, is still limited. For example, *CTTNBP2* is an autism-related gene mainly expressed in neurons and highly enriched in the dendritic spine. It is involved in stabilizing microtubules and controlling dendritic spine formation [49]. Upregulation of *CTTNBP2* promotes the cancerous growth of the ovary [50]. KIF18A is an MT depolymerizing kinesin that ensures chromosome stability during mitosis by inhibiting MT dynamics without disrupting their stability [51]. *KIF18A* upregulation contributes to the malignant progression and unfavorable prognosis of various types of cancer, including breast, hepatocellular, clear cell renal, prostate, esophageal carcinomas, and lung adenocarcinoma, especially glioma [52,53,54,55,56,57,58,59,60,61]. *SLAIN2* promotes the nucleation and elongation of cytoplasmic MT and suppresses their catastrophes, which is necessary for maintaining the normal structure of the cytoskeleton [62]. Increased *SLAIN2* expression encourages the invasion of mesenchymal cells in three-dimensional culture and mouse cancer models. In addition, it promotes MT migration and increases the invasion as well as metastasis of colon cancer, leading to a poor prognosis [63,64]. *SRCIN1* plays a role in synaptic maintenance and neurotransmitter release. Moreover, as an anti-tumor molecule, its downregulation stimulates the spread, migration, and invasion of lung, liver, breast, and gastric cancers and neuroblastoma, thereby affecting their prognosis and treatment [65,66,67,68,69]. *TTBK2* inhibits the activity of the microtubule depolymerase of KIF2A through phosphorylation, thus promoting the migration of cervical cancer cells. Additionally, it also has a crucial function in cilia formation [70,71]. *TTBK2* downregulation sensitizes melanoma cells and renal cells to sunitinib [72]. *NAV1* is essential for neurogenesis and participates in neuronal migration [73]. *TRIO* promotes actin remodeling, cell growth and migration, which is necessary for maintaining proper axonal growth and MT network stability [74,75,76]. However, the correlation between *NAV1*, *TRIO*, and cancer has not been well investigated. Our study found that compared with normal tissues, *CTTNBP2*, *KIF18A*, *NAV1*, *SLAIN2*, and *TRIO* were upregulated in glioma, while *SRCIN1* and *TTBK2* were downregulated. Moreover, *TTBK2*, *NAV1*, and *CTTNBP2* were protective factors for glioma, while *SRCIN1*, *TRIO*, *KIF18A*, and *SLAIN2* were risk factors. In general, the signature composed of these seven genes is a risk factor for glioma development, and with an increase in the risk score, glioma was more inclined to malignant progression. Therefore, we also designed a nomogram model containing clinical features and risk scores. Utilizing the TCGA and CGGA datasets, we further confirmed that our construction is the best prognostic predictor for glioma patients.

In addition to regulating MT plus end dynamics to affect glioma, this research also studied the connection between the clusters, risk groups, and TME infiltration. Consistent with the cluster results, the risk scores were strongly connected to the TME scores, especially the immune score, indicating that they may form an anti-tumor immune microenvironment. Nonetheless, a growing number of investigations have pointed out that different immune cells exhibit different immune functions, which may promote or inhibit the antitumor immune response. CD4 Th2, CD4 Treg, and macrophage M2 are known to be representative immunosuppressive cells that promote cancer growth [77,78,79]; hence, we further studied these cells. The risk score was strongly connected to the CD4 Th2 cell infiltration and negatively connected to the CD4 Treg cell infiltration by the ssGSEA algorithm. In addition to these two consistent correlations, the xCell algorithm also revealed that the risk score was strongly connected to macrophage M2, implying that the anti-tumor immune microenvironment is accompanied by the tumor-promoting immune microenvironment. In general, we speculated that in the signature-associated glioma patients, with the malignant progression of the disease, the increased risk score leading to an unfavorable prognosis is related to the suppressive immune microenvironment.

As a novel and promising treatment method, immunotherapy for glioma has attracted increasing attention. Our results indicated that the risk signature was strongly connected to the immunoinhibitors and TMB, and negatively connected to the MSI in glioma. In addition, the ICB therapy prediction confirmed that the treatment effect was inferior in the high-risk patients compared to those at low risk. Combined with the clinical factors, these findings revealed that ICB therapy may be more efficient for LGG patients than GBM patients. Therefore, according to the current results, our signature could be utilized as a reliable biomarker for immunotherapy in glioma patients at different stages of progression. Additionally, we found several associations between the IC50 of chemical drugs and signature gene expression, representing another potential molecular target for glioma chemotherapy that may help to predict chemotherapy resistance.

Inevitably, this research also has certain drawbacks. Although the information was gathered from several public databases, additional experimental verification is needed. In addition, the data showed some unexpected results, such as the negative correlation between the cancer stemness and risk score. However, the progression, prognosis and treatment of glioma are inherently related to multiple factors, and the contribution of a single factor to the above-mentioned process is often limited. Therefore, further research in larger-scale and multicenter clinical cohorts is needed to overcome these inconsistent results.

## 5. Conclusions

In summary, this work represents the first to construct and validate an MPERG-based prognostic signature associated with the TME as a reliable and independent prognostic biomarker to provide personalized choices of immunotherapy and chemotherapy for glioma patients.

## Figures and Tables

**Table 1:** The basic information of the 46 MPEPGs.

Gene Name	Protein Name	Binding Mode
*AMER2 (FAM123A)*	APC membrane recruitment protein 2	Via MAPRE1/2/3
*APC (DP2.5)*	Adenomatous polyposis coli protein	Autonomous or via MAPRE1/2/3
*APC2 (APCL)*	Adenomatous polyposis coli protein 2	Via MAPRE1/2/3
*CDK5RAP2 (CEP215)*	CDK5 regulatory subunit-associated protein 2	Via MAPRE1/2/3
*CEP104 (KIAA0562)*	Centrosomal protein of 104 kDa	Via MAPRE1/2/3
*CKAP5 (ch-TOG)*	Cytoskeleton-associated protein 5	Autonomous
*CLASP1 (MAST1)*	CLIP-associating protein 1	Via MAPRE1/2/3
*CLASP2 (KIAA0627)*	CLIP-associating protein 2	Via MAPRE1/2/3
*CLIP1 (CYLN1, CLIP-170)*	CAP-Gly domain-containing linker protein 1	Via MAPRE1/2/3
*CLIP2 (CYLN2, CLIP-115)*	CAP-Gly domain-containing linker protein 2	Via MAPRE1/2/3
*CLIP3 (CLIPR59)*	CAP-Gly domain-containing linker protein 3	Via MAPRE1/2/3
*CLIP4 (RSNL2)*	CAP-Gly domain-containing linker protein 4	Via MAPRE1/2/3
*CTTNBP2 (CORTBP2)*	Cortactin-binding protein 2	Via MAPRE1/2/3
*DCTN1 (p150glued)*	Dynactin subunit 1	Via MAPRE1/2/3
*DST*	Dystonin	Via MAPRE1/2/3
*FBXW11*	F-box/WD repeat-containing protein 11	Via MAPRE1/2/3
*FILIP1 (KIAA1275)*	Filamin-A-interacting protein 1	Via MAPRE1/2/3
*GAS2L1 (GAR22)*	GAS2-like protein 1	Via MAPRE1/2/3
*GAS2L2 (GAR17)*	GAS2-like protein 2	Via MAPRE1/2/3
*KIF11 (Eg5)*	Kinesin-like protein KIF11	Via MAPRE1/2/3
*KIF18A*	Kinesin-like protein KIF18A	Via MAPRE1/2/3
*KIF18B*	Kinesin-like protein KIF18B	Via MAPRE1/2/3
*KIF2C (MCAK)*	Kinesin-like protein KIF2C	Via MAPRE1/2/3
*KNSTRN (SKAP)*	Small kinetochore-associated protein	Via MAPRE1/2/3
*MACF1 (ACF7)*	Microtubule-actin cross-linking factor1	Via MAPRE1/2/3
*MAPRE1 (EB1)*	Microtubule-associated protein RP/EB family member 1	Autonomous
*MAPRE2 (EB2)*	Microtubule-associated protein RP/EB family member 2	Via MAPRE1/2/3
*MAPRE3 (EB3)*	Microtubule-associated protein RP/EB family member 3	Via MAPRE1/2/3
*NAV1 (POMFIL3)*	Neuron navigator 1	Via MAPRE1/2/3
*NAV2 (POMFIL2)*	Neuron navigator 2	Via MAPRE1/2/3
*NAV3 (POMFIL1)*	Neuron navigator 3	Via MAPRE1/2/3
*NCKAP5 (ERIH, NAP5)*	Nck-associated protein 5	Via MAPRE1/2/3
*NCKAP5L (CEP169)*	Nck-associated protein 5-like	Via MAPRE1/2/3
*PAFAH1B1 (LIS1)*	Lissencephaly-1 homolog	Via CLIP-1
*PPP1R13L (iASPP)*	RelA-associated inhibitor	Via MAPRE1/2/3
*PSRC1 (DDA3)*	Proline/serine-rich coiled-coil protein 1	Via MAPRE1/2/3
*SLAIN1 (C13orf32)*	SLAIN motif-containing protein 1	Via MAPRE1/2/3
*SLAIN2 (KIAA1458)*	SLAIN motif-containing protein 2	Via MAPRE1/2/3
*SPAG5 (Astrin)*	Sperm-associated antigen 5	Via MAPRE1/2/3
*SRCIN1 (P140)*	SRC kinase signaling inhibitor 1	Via MAPRE1/2/3
*STIM1 (GOK)*	Stromal interaction molecule 1	Via MAPRE1/2/3
*SYBU (GOLSYN)*	Syntabulin	Via MAPRE1/2/3
*TACC3 (ERIC1)*	Transforming acidic coiled-coil-containing protein 3	Unclear
*TRIO (ARHGEF23)*	Triple functional domain protein	Via MAPRE1/2/3
*TTBK1 (BDTK)*	Tau-tubulin kinase 1	Via MAPRE1/2/3
*TTBK2 (KIAA0847)*	Tau-tubulin kinase 2	Via MAPRE1/2/3

**Table 2:** Baseline datasheet of the risk grouping comparison under different clinical factors in glioma from the TCGA database.

Characteristic	Low Risk (334)	High Risk (335)	*p*
WHO grade, n (%)			<0.001 ***
G2	169 (27.6%)	46 (7.2%)	
G3	127 (20.8%)	110 (18%)	
G4	1 (0.2%)	159 (26%)	
IDH status, n (%)			<0.001 ***
WT	10 (1.5%)	227 (34.4%)	
Mut	322 (48.8%)	101 (15.3%)	
1p/19q codeletion, n (%)			<0.001 ***
Codel	158 (23.8%)	9 (1.4%)	
Non-codel	176 (26.5%)	320 (48.3%)	
Histological type, n (%)			<0.001 ***
Astrocytoma	88 (13.2%)	104 (15.5%)	
Glioblastoma	1 (0.1%)	159 (23.8%)	
Oligoastrocytoma	86 (12.9%)	42 (6.3%)	
Oligodendroglioma	159 (23.8%)	30 (4.5%)	
Primary therapy outcome (PTO), n (%)			0.001 **
Progressive disease (PD)	53 (12%)	50 (11.3%)	
Stable disease (SD)	102 (23%)	42 (9.5%)	
Partial response (PR)	42 (9.5%)	20 (4.5%)	
Complete response (CR)	100 (22.6%)	34 (7.7%)	
Gender, n (%)			0.331
Female	148 (22.1%)	135 (20.2%)	
Male	186 (27.8%)	200 (29.9%)	
Race, n (%)			0.200
Asian	6 (0.9%)	7 (1.1%)	
Black or African American	11 (1.7%)	21 (3.2%)	
White	309 (47%)	303 (46.1%)	
Age, n (%)			<0.001 ***
≤60	305 (45.6%)	225 (33.6%)	
>60	29 (4.3%)	110 (16.4%)	

** *p* < 0.01, *** *p* < 0.001.

**Table 3:** Baseline datasheet of the risk grouping comparison under different clinical factors in glioma from the CGGA database.

Characteristic	Low Risk (485)	High Risk (485)	*p*
WHO grade, n (%)			<0.001 ***
G2	39 (4%)	231 (23.9%)	
G3	131 (13.6%)	191 (19.8%)	
G4	311 (32.2%)	63 (6.5%)	
IDH status, n (%)			<0.001 ***
WT	346 (37.6%)	75 (8.1%)	
Mutant	132 (14.3%)	368 (40%)	
1p/19q codeletion, n (%)			<0.001 ***
Codel	14 (1.6%)	185 (20.6%)	
Non-codel	439 (49%)	258 (28.8%)	
Primary-recurrent-secondary (PRS) type, n (%)			<0.001 ***
Primary	273 (28.3%)	353 (36.5%)	
Secondary	23 (2.4%)	6 (0.6%)	
Recurrent	185 (19.2%)	126 (13%)	
Age, n (%)			<0.001 ***
≤60	414 (42.7%)	463 (47.8%)	
>60	70 (7.2%)	22 (2.3%)	
Gender, n (%)			0.117
Female	187 (19.3%)	212 (21.9%)	
Male	298 (30.7%)	273 (28.1%)	

*** *p* < 0.001.

## Data Availability

The data presented in this study are available in the article and Supplementary Materials.

## Supplementary Materials

The following supporting information can be downloaded at https://www.mdpi.com/article/10.3390/biology12030488/s1, Figure S1: Glioma patients with DEMPERGs were distinguished into three subgroups via unsupervised consensus clustering; Figure S2: Identification of the prognostic-related DEMPERGs in glioma. Figure S3: Different TME infiltrations between the two risk groups in glioma; Table S1. Other gene mutation frequencies in the MPERG mutant group and non-mutant group. Table S2: The CNA frequency of the MPERGs; Table S3: Correlations between the MPERG expression and CNA, DNA methylation in the LGG and GBM from cBioPortal; Table S4: Correlations among the MPERGs from the TCGA database; Table S5: Node degree of the MPERG interaction network from the STRING database; Table S6: GO and KEGG analyses of 46 MPERGs; Table S7: GSEA analyses of the cluster-associated DEGs; Table S8: Correlations between the risk signature and TME infiltration using the ssGSEA and ESTIMATE algorithm in glioma from the TCGA database; Table S9: Correlations between the risk signature and TME infiltration using the xCELL algorithm in glioma from the TCGA database; Table S10: Correlations between the risk signature and immunoinhibitors in glioma from the TCGA database; Table S11: Correlations between the risk signature and TMB and MSI scores in glioma from the TCGA database.

## References

[1] Sung H., Ferlay J., Siegel R.L., Laversanne M., Soerjomataram I., Jemal A., Bray F. (2021). Global Cancer Statistics 2020: GLOBOCAN Estimates of Incidence and Mortality Worldwide for 36 Cancers in 185 Countries. CA Cancer J. Clin..

[2] Chen F., Wendl M.C., Wyczalkowski M.A., Bailey M.H., Li Y., Ding L. (2021). Moving pan-cancer studies from basic research toward the clinic. Nat. Cancer.

[3] Louis D.N., Perry A., Reifenberger G., von Deimling A., Figarella-Branger D., Cavenee W.K., Ohgaki H., Wiestler O.D., Kleihues P., Ellison D.W. (2016). The 2016 World Health Organization Classification of Tumors of the Central Nervous System: A summary. Acta Neuropathol..

[4] Liu S.L., Sun X.S., Chen Q.Y., Liu Z.X., Bian L.J., Yuan L., Xiao B.B., Lu Z.J., Li X.Y., Yan J.J. (2022). Development and validation of a transcriptomics-based gene signature to predict distant metastasis and guide induction chemotherapy in locoregionally advanced nasopharyngeal carcinoma. Eur. J. Cancer.

[5] Bruni D., Angell H.K., Galon J. (2020). The immune contexture and Immunoscore in cancer prognosis and therapeutic efficacy. Nat. Rev. Cancer.

[6] Kelly W.J., Giles A.J., Gilbert M. (2020). T lymphocyte-targeted immune checkpoint modulation in glioma. J. Immunother. Cancer.

[7] Xu S., Tang L., Li X., Fan F., Liu Z. (2020). Immunotherapy for glioma: Current management and future application. Cancer Lett..

[8] Jordan M.A., Wilson L. (2004). Microtubules as a target for anticancer drugs. Nat. Rev. Cancer.

[9] Karahalil B., Yardim-Akaydin S., Nacak Baytas S. (2019). An overview of microtubule targeting agents for cancer therapy. Arh. Hig. Rada Toksikol..

[10] Meiring J.C.M., Shneyer B.I., Akhmanova A. (2020). Generation and regulation of microtubule network asymmetry to drive cell polarity. Curr. Opin. Cell Biol..

[11] Howard J., Hyman A.A. (2003). Dynamics and mechanics of the microtubule plus end. Nature.

[12] van de Willige D., Hoogenraad C.C., Akhmanova A. (2016). Microtubule plus-end tracking proteins in neuronal development. Cell Mol. Life Sci..

[13] Aher A., Akhmanova A. (2018). Tipping microtubule dynamics, one protofilament at a time. Curr. Opin. Cell. Biol..

[14] Borys F., Joachimiak E., Krawczyk H., Fabczak H. (2020). Intrinsic and Extrinsic Factors Affecting Microtubule Dynamics in Normal and Cancer Cells. Molecules.

[15] Wattanathamsan O., Pongrakhananon V. (2022). Emerging role of microtubule-associated proteins on cancer metastasis. Front. Pharmacol..

[16] Vivian J., Rao A.A., Nothaft F.A., Ketchum C., Armstrong J., Novak A., Pfeil J., Narkizian J., Deran A.D., Musselman-Brown A. (2017). Toil enables reproducible, open source, big biomedical data analyses. Nat. Biotechnol..

[17] Liu J., Lichtenberg T., Hoadley K.A., Poisson L.M., Lazar A.J., Cherniack A.D., Kovatich A.J., Benz C.C., Levine D.A., Lee A.V. (2018). An Integrated TCGA Pan-Cancer Clinical Data Resource to Drive High-Quality Survival Outcome Analytics. Cell.

[18] Zhao Z., Zhang K.N., Wang Q., Li G., Zeng F., Zhang Y., Wu F., Chai R., Wang Z., Zhang C. (2021). Chinese Glioma Genome Atlas (CGGA): A Comprehensive Resource with Functional Genomic Data from Chinese Glioma Patients. Genom. Proteom. Bioinform..

[19] Cerami E., Gao J., Dogrusoz U., Gross B.E., Sumer S.O., Aksoy B.A., Jacobsen A., Byrne C.J., Heuer M.L., Larsson E. (2012). The cBio cancer genomics portal: An open platform for exploring multidimensional cancer genomics data. Cancer Discov..

[20] Gao J., Aksoy B.A., Dogrusoz U., Dresdner G., Gross B., Sumer S.O., Sun Y., Jacobsen A., Sinha R., Larsson E. (2013). Integrative analysis of complex cancer genomics and clinical profiles using the cBioPortal. Sci. Signal..

[21] Mayakonda A., Lin D.C., Assenov Y., Plass C., Koeffler H.P. (2018). Maftools: Efficient and comprehensive analysis of somatic variants in cancer. Genome Res..

[22] Gu Z., Eils R., Schlesner M. (2016). Complex heatmaps reveal patterns and correlations in multidimensional genomic data. Bioinformatics.

[23] Szklarczyk D., Gable A.L., Lyon D., Junge A., Wyder S., Huerta-Cepas J., Simonovic M., Doncheva N.T., Morris J.H., Bork P. (2019). STRING v11: Protein-protein association networks with increased coverage, supporting functional discovery in genome-wide experimental datasets. Nucleic Acids Res..

[24] Yi L., Wu G., Guo L., Zou X., Huang P. (2020). Comprehensive Analysis of the PD-L1 and Immune Infiltrates of m(6)A RNA Methylation Regulators in Head and Neck Squamous Cell Carcinoma. Mol. Ther. Nucleic Acids.

[25] Li Y., Xiao J., Bai J., Tian Y., Qu Y., Chen X., Wang Q., Li X., Zhang Y., Xu J. (2019). Molecular characterization and clinical relevance of m(6)A regulators across 33 cancer types. Mol. Cancer.

[26] Zhang Z., Lin E., Zhuang H., Xie L., Feng X., Liu J., Yu Y. (2020). Construction of a novel gene-based model for prognosis prediction of clear cell renal cell carcinoma. Cancer Cell. Int..

[27] Love M.I., Huber W., Anders S. (2014). Moderated estimation of fold change and dispersion for RNA-seq data with DESeq2. Genome Biol..

[28] Yu G., Wang L.G., Han Y., He Q.Y. (2012). clusterProfiler: An R package for comparing biological themes among gene clusters. OMICS.

[29] Subramanian A., Tamayo P., Mootha V.K., Mukherjee S., Ebert B.L., Gillette M.A., Paulovich A., Pomeroy S.L., Golub T.R., Lander E.S. (2005). Gene set enrichment analysis: A knowledge-based approach for interpreting genome-wide expression profiles. Proc. Natl. Acad. Sci. USA.

[30] Vickers A.J., Elkin E.B. (2006). Decision curve analysis: A novel method for evaluating prediction models. Med. Decis. Mak..

[31] Bindea G., Mlecnik B., Tosolini M., Kirilovsky A., Waldner M., Obenauf A.C., Angell H., Fredriksen T., Lafontaine L., Berger A. (2013). Spatiotemporal dynamics of intratumoral immune cells reveal the immune landscape in human cancer. Immunity.

[32] Hanzelmann S., Castelo R., Guinney J. (2013). GSVA: Gene set variation analysis for microarray and RNA-seq data. BMC Bioinform..

[33] Yoshihara K., Shahmoradgoli M., Martinez E., Vegesna R., Kim H., Torres-Garcia W., Trevino V., Shen H., Laird P.W., Levine D.A. (2013). Inferring tumour purity and stromal and immune cell admixture from expression data. Nat. Commun..

[34] Aran D., Hu Z., Butte A.J. (2017). xCell: Digitally portraying the tissue cellular heterogeneity landscape. Genome Biol..

[35] Thorsson V., Gibbs D.L., Brown S.D., Wolf D., Bortone D.S., Ou Yang T.H., Porta-Pardo E., Gao G.F., Plaisier C.L., Eddy J.A. (2018). The Immune Landscape of Cancer. Immunity.

[36] Samstein R.M., Lee C.H., Shoushtari A.N., Hellmann M.D., Shen R., Janjigian Y.Y., Barron D.A., Zehir A., Jordan E.J., Omuro A. (2019). Tumor mutational load predicts survival after immunotherapy across multiple cancer types. Nat. Genet..

[37] Bonneville R., Krook M.A., Kautto E.A., Miya J., Wing M.R., Chen H.Z., Reeser J.W., Yu L., Roychowdhury S. (2017). Landscape of Microsatellite Instability Across 39 Cancer Types. JCO Precis. Oncol..

[38] Malta T.M., Sokolov A., Gentles A.J., Burzykowski T., Poisson L., Weinstein J.N., Kaminska B., Huelsken J., Omberg L., Gevaert O. (2018). Machine Learning Identifies Stemness Features Associated with Oncogenic Dedifferentiation. Cell.

[39] Jiang P., Gu S., Pan D., Fu J., Sahu A., Hu X., Li Z., Traugh N., Bu X., Li B. (2018). Signatures of T cell dysfunction and exclusion predict cancer immunotherapy response. Nat. Med..

[40] Wang Q., Li M., Yang M., Yang Y., Song F., Zhang W., Li X., Chen K. (2020). Analysis of immune-related signatures of lung adenocarcinoma identified two distinct subtypes: Implications for immune checkpoint blockade therapy. Aging.

[41] Liu C.J., Hu F.F., Xia M.X., Han L., Zhang Q., Guo A.Y. (2018). GSCALite: A web server for gene set cancer analysis. Bioinformatics.

[42] Lansbergen G., Akhmanova A. (2006). Microtubule plus end: A hub of cellular activities. Traffic.

[43] Liberzon A., Birger C., Thorvaldsdottir H., Ghandi M., Mesirov J.P., Tamayo P. (2015). The Molecular Signatures Database (MSigDB) hallmark gene set collection. Cell Syst..

[44] Mendiratta G., Ke E., Aziz M., Liarakos D., Tong M., Stites E.C. (2021). Cancer gene mutation frequencies for the U.S. population. Nat. Commun..

[45] Eckel-Passow J.E., Lachance D.H., Molinaro A.M., Walsh K.M., Decker P.A., Sicotte H., Pekmezci M., Rice T., Kosel M.L., Smirnov I.V. (2015). Glioma Groups Based on 1p/19q, IDH, and TERT Promoter Mutations in Tumors. N. Engl. J. Med..

[46] Tao Z., Wang S., Wu C., Wu T., Zhao X., Ning W., Wang G., Wang J., Chen J., Diao K. (2023). The repertoire of copy number alteration signatures in human cancer. Brief. Bioinform..

[47] Nishiyama A., Nakanishi M. (2021). Navigating the DNA methylation landscape of cancer. Trends Genet..

[48] Singh A., Settleman J. (2010). EMT, cancer stem cells and drug resistance: An emerging axis of evil in the war on cancer. Oncogene.

[49] Shih P.Y., Lee S.P., Chen Y.K., Hsueh Y.P. (2014). Cortactin-binding protein 2 increases microtubule stability and regulates dendritic arborization. J. Cell Sci..

[50] Si L., Chen J., Yang S., Liu Z., Chen Y., Peng M., Jia Y. (2020). lncRNA HEIH accelerates cell proliferation and inhibits cell senescence by targeting miR-3619-5p/CTTNBP2 axis in ovarian cancer. Menopause.

[51] Marquis C., Fonseca C.L., Queen K.A., Wood L., Vandal S.E., Malaby H.L.H., Clayton J.E., Stumpff J. (2021). Chromosomally unstable tumor cells specifically require KIF18A for proliferation. Nat. Commun..

[52] Zhang C., Zhu C., Chen H., Li L., Guo L., Jiang W., Lu S.H. (2010). Kif18A is involved in human breast carcinogenesis. Carcinogenesis.

[53] Liao W., Huang G., Liao Y., Yang J., Chen Q., Xiao S., Jin J., He S., Wang C. (2014). High KIF18A expression correlates with unfavorable prognosis in primary hepatocellular carcinoma. Oncotarget.

[54] Chen Q.I., Cao B., Nan N., Wang Y.U., Zhai X.U., Li Y., Chong T. (2016). Elevated expression of KIF18A enhances cell proliferation and predicts poor survival in human clear cell renal carcinoma. Exp. Ther. Med..

[55] Luo W., Liao M., Liao Y., Chen X., Huang C., Fan J., Liao W. (2018). The role of kinesin KIF18A in the invasion and metastasis of hepatocellular carcinoma. World J. Surg. Oncol..

[56] Alfarsi L.H., Elansari R., Toss M.S., Diez-Rodriguez M., Nolan C.C., Ellis I.O., Rakha E.A., Green A.R. (2019). Kinesin family member-18A (KIF18A) is a predictive biomarker of poor benefit from endocrine therapy in early ER+ breast cancer. Breast Cancer Res. Treat..

[57] Chen F.T., Zhong F.K. (2019). Kinesin Family Member 18A (KIF18A) Contributes to the Proliferation, Migration, and Invasion of Lung Adenocarcinoma Cells In Vitro and In Vivo. Dis. Markers.

[58] Zhang H., Shen T., Zhang Z., Li Y., Pan Z. (2019). Expression of KIF18A Is Associated with Increased Tumor Stage and Cell Proliferation in Prostate Cancer. Med. Sci. Monit..

[59] Zhong Y., Jiang L., Lin H., Li X., Long X., Zhou Y., Li B., Li Z. (2019). Overexpression of KIF18A promotes cell proliferation, inhibits apoptosis, and independently predicts unfavorable prognosis in lung adenocarcinoma. IUBMB Life.

[60] Qian L.X., Cao X., Du M.Y., Ma C.X., Zhu H.M., Peng Y., Hu X.Y., He X., Yin L. (2021). KIF18A knockdown reduces proliferation, migration, invasion and enhances radiosensitivity of esophageal cancer. Biochem. Biophys. Res. Commun..

[61] Tao B.Y., Liu Y.Y., Liu H.Y., Zhang Z.H., Guan Y.Q., Wang H., Shi Y., Zhang J. (2022). Prognostic Biomarker KIF18A and Its Correlations With Immune Infiltrates and Mitosis in Glioma. Front. Genet..

[62] van der Vaart B., Manatschal C., Grigoriev I., Olieric V., Gouveia S.M., Bjelic S., Demmers J., Vorobjev I., Hoogenraad C.C., Steinmetz M.O. (2011). SLAIN2 links microtubule plus end-tracking proteins and controls microtubule growth in interphase. J. Cell Biol..

[63] Bouchet B.P., Noordstra I., van Amersfoort M., Katrukha E.A., Ammon Y.C., Ter Hoeve N.D., Hodgson L., Dogterom M., Derksen P.W.B., Akhmanova A. (2016). Mesenchymal Cell Invasion Requires Cooperative Regulation of Persistent Microtubule Growth by SLAIN2 and CLASP1. Dev. Cell.

[64] Zhuang M., Zhao S., Jiang Z., Wang S., Sun P., Quan J., Yan D., Wang X. (2019). MALAT1 sponges miR-106b-5p to promote the invasion and metastasis of colorectal cancer via SLAIN2 enhanced microtubules mobility. eBioMedicine.

[65] Grasso S., Cangelosi D., Chapelle J., Alzona M., Centonze G., Lamolinara A., Salemme V., Angelini C., Morellato A., Saglietto A. (2020). The SRCIN1/p140Cap adaptor protein negatively regulates the aggressiveness of neuroblastoma. Cell. Death Differ..

[66] Zhang Y., Yuan J., Guo M., Xiang R., Wang X., Xie T., Zhuang X., Li Q., Lai Q. (2022). miR-657 Targets SRCIN1 via the Slug Pathway to Promote NSCLC Tumor Growth and EMT Induction. Dis. Markers.

[67] Bai P.S., Hou P., Kong Y. (2018). Hepatitis B virus promotes proliferation and metastasis in male Chinese hepatocellular carcinoma patients through the LEF-1/miR-371a-5p/SRCIN1/pleiotrophin/Slug pathway. Exp. Cell Res..

[68] Xu X., Wang W., Su N., Zhu X., Yao J., Gao W., Hu Z., Sun Y. (2015). miR-374a promotes cell proliferation, migration and invasion by targeting SRCIN1 in gastric cancer. FEBS Lett..

[69] Yang F., Luo L.J., Zhang L., Wang D.D., Yang S.J., Ding L., Li J., Chen D., Ma R., Wu J.Z. (2017). MiR-346 promotes the biological function of breast cancer cells by targeting SRCIN1 and reduces chemosensitivity to docetaxel. Gene.

[70] Nguyen A., Goetz S.C. (2022). TTBK2 controls cilium stability by regulating distinct modules of centrosomal proteins. Mol. Biol. Cell..

[71] Watanabe T., Kakeno M., Matsui T., Sugiyama I., Arimura N., Matsuzawa K., Shirahige A., Ishidate F., Nishioka T., Taya S. (2015). TTBK2 with EB1/3 regulates microtubule dynamics in migrating cells through KIF2A phosphorylation. J. Cell Biol..

[72] Bender C., Ullrich A. (2012). PRKX, TTBK2 and RSK4 expression causes Sunitinib resistance in kidney carcinoma- and melanoma-cell lines. Int. J. Cancer.

[73] Powers R.M., Daza R., Koehler A.E., Courchet J., Calabrese B., Hevner R.F., Halpain S. (2022). Growth cone macropinocytosis of neurotrophin receptor and neuritogenesis are regulated by neuron navigator 1. Mol. Biol. Cell.

[74] van Haren J., Boudeau J., Schmidt S., Basu S., Liu Z., Lammers D., Demmers J., Benhari J., Grosveld F., Debant A. (2014). Dynamic microtubules catalyze formation of navigator-TRIO complexes to regulate neurite extension. Curr. Biol..

[75] Seipel K., Medley Q.G., Kedersha N.L., Zhang X.A., O’Brien S.P., Serra-Pages C., Hemler M.E., Streuli M. (1999). Trio amino-terminal guanine nucleotide exchange factor domain expression promotes actin cytoskeleton reorganization, cell migration and anchorage-independent cell growth. J. Cell Sci..

[76] Deinhardt K., Kim T., Spellman D.S., Mains R.E., Eipper B.A., Neubert T.A., Chao M.V., Hempstead B.L. (2011). Neuronal growth cone retraction relies on proneurotrophin receptor signaling through Rac. Sci. Signal..

[77] Hirahara K., Nakayama T. (2016). CD4+ T-cell subsets in inflammatory diseases: Beyond the Th1/Th2 paradigm. Int. Immunol..

[78] Scott E.N., Gocher A.M., Workman C.J., Vignali D.A.A. (2021). Regulatory T Cells: Barriers of Immune Infiltration Into the Tumor Microenvironment. Front. Immunol..

[79] Liu X., Liu Y., Qi Y., Huang Y., Hu F., Dong F., Shu K., Lei T. (2022). Signal Pathways Involved in the Interaction Between Tumor-Associated Macrophages/TAMs and Glioblastoma Cells. Front. Oncol..

