# Supplementary material for: Dynamic Regulation Genes at Microtubule Plus Ends: A Novel Class of Glioma Biomarkers
Source: Biology (Basel). 2023 Mar 22;12(3):488. doi: 10.3390/biology12030488 (PMC10045452; doi:10.3390/biology12030488)
Supplement: Supplementary file 1 [file biology-12-00488-s001.zip › Supplementary Figures.pdf]

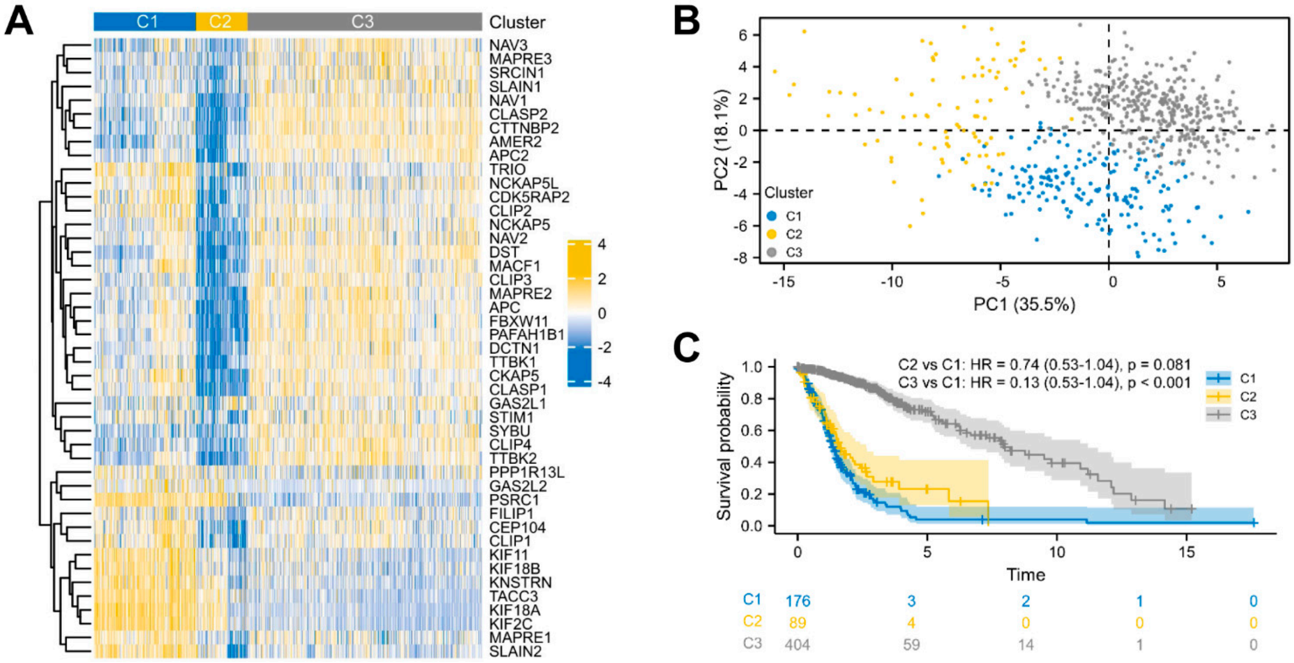

Figure S1. Glioma patients with DEMPERGs were distinguished into three subgroups by unsupervised consensus clustering. Related to Figure 4. (**A**) Complex heatmap of DEMPERG expression. (**B**) PCA plot. (**C**) KM OS plot.

A

Characteristics	HR (95% CI) Uni-OS	P value	HR (95% CI) Uni -DSS	P value	HR (95% CI) Uni -PFI	P value
AMER2	0.568 (0.524-0.615)	<0.001	0.562 (0.515-0.612)	<0.001	0.625 (0.580-0.674)	<0.001
APC	0.564 (0.487-0.654)	<0.001	0.573 (0.490-0.670)	<0.001	0.630 (0.546-0.727)	<0.001
APC2	0.551 (0.496-0.612)	<0.001	0.528 (0.470-0.593)	<0.001	0.598 (0.541-0.661)	<0.001
CDK5RAP2	0.714 (0.572-0.892)	0.003	0.685 (0.542-0.865)	0.001	0.742 (0.602-0.913)	0.005
CEP104	1.287 (0.982-1.687)	0.067	1.304 (0.982-1.732)	0.067	1.429 (1.121-1.821)	0.004
CKAP5	0.668 (0.545-0.819)	<0.001	0.650 (0.525-0.804)	<0.001	0.720 (0.593-0.874)	<0.001
CLASP1	0.565 (0.472-0.676)	<0.001	0.558 (0.462-0.674)	<0.001	0.583 (0.490-0.695)	<0.001
CLASP2	0.381 (0.329-0.443)	<0.001	0.373 (0.318-0.437)	<0.001	0.465 (0.408-0.531)	<0.001
CLIP1	1.335 (1.060-1.682)	0.014	1.346 (1.055-1.717)	0.017	1.415 (1.149-1.743)	0.001
CLIP2	0.777 (0.652-0.927)	0.005	0.752 (0.626-0.904)	0.002	0.819 (0.686-0.977)	0.027
CLIP3	0.473 (0.404-0.554)	<0.001	0.467 (0.396-0.551)	<0.001	0.509 (0.434-0.597)	<0.001
CTTNBP2	0.519 (0.468-0.576)	<0.001	0.515 (0.462-0.575)	<0.001	0.569 (0.516-0.628)	<0.001
DST	0.512 (0.458-0.572)	<0.001	0.507 (0.451-0.571)	<0.001	0.581 (0.523-0.646)	<0.001
FBXW11	0.434 (0.351-0.537)	<0.001	0.437 (0.349-0.547)	<0.001	0.491 (0.401-0.602)	<0.001
FILIP1	0.892 (0.711-1.119)	0.322	0.905 (0.713-1.149)	0.414	0.917 (0.750-1.120)	0.395
GAS2L1	0.594 (0.483-0.731)	<0.001	0.604 (0.482-0.757)	<0.001	0.672 (0.555-0.813)	<0.001
KIF11	1.513 (1.362-1.680)	<0.001	1.511 (1.351-1.690)	<0.001	1.325 (1.207-1.455)	<0.001
KIF18A	2.488 (2.199-2.816)	<0.001	2.569 (2.249-2.933)	<0.001	1.964 (1.769-2.179)	<0.001
KIF18B	1.374 (1.251-1.510)	<0.001	1.365 (1.235-1.509)	<0.001	1.292 (1.188-1.406)	<0.001
KIF2C	1.734 (1.599-1.880)	<0.001	1.739 (1.597-1.894)	<0.001	1.510 (1.409-1.618)	<0.001
KNSTRN	2.100 (1.788-2.467)	<0.001	2.083 (1.758-2.469)	<0.001	1.642 (1.419-1.899)	<0.001
MAPRE1	1.426 (1.101-1.848)	0.007	1.463 (1.111-1.927)	0.007	1.303 (1.041-1.630)	0.021
MAPRE2	0.496 (0.403-0.610)	<0.001	0.489 (0.394-0.607)	<0.001	0.524 (0.431-0.637)	<0.001
NAV1	0.547 (0.481-0.624)	<0.001	0.534 (0.466-0.613)	<0.001	0.610 (0.541-0.687)	<0.001
NAV2	0.705 (0.610-0.814)	<0.001	0.701 (0.602-0.817)	<0.001	0.747 (0.655-0.852)	<0.001
NCKAP5	0.631 (0.529-0.753)	<0.001	0.640 (0.530-0.772)	<0.001	0.788 (0.673-0.922)	0.003
NCKAP5L	0.824 (0.647-1.048)	0.115	0.770 (0.598-0.991)	0.042	0.782 (0.628-0.972)	0.027
PAFAH1B1	0.416 (0.339-0.510)	<0.001	0.405 (0.327-0.502)	<0.001	0.437 (0.359-0.533)	<0.001
PPP1R13L	1.269 (1.114-1.447)	<0.001	1.277 (1.113-1.465)	<0.001	1.187 (1.053-1.338)	0.005
PSRC1	2.096 (1.898-2.316)	<0.001	2.102 (1.893-2.335)	<0.001	1.822 (1.673-1.985)	<0.001
SLAIN1	0.621 (0.541-0.713)	<0.001	0.613 (0.526-0.715)	<0.001	0.726 (0.645-0.817)	<0.001
SLAIN2	3.770 (2.845-4.995)	<0.001	4.092 (3.032-5.524)	<0.001	2.781 (2.180-3.547)	<0.001
STIM1	0.931 (0.744-1.165)	0.533	0.945 (0.746-1.199)	0.643	0.940 (0.771-1.147)	0.542
SYBU	0.555 (0.492-0.626)	<0.001	0.539 (0.471-0.617)	<0.001	0.659 (0.589-0.737)	<0.001
TACC3	1.561 (1.464-1.665)	<0.001	1.552 (1.450-1.661)	<0.001	1.516 (1.411-1.629)	<0.001
TRIO	1.425 (1.229-1.652)	<0.001	1.413 (1.204-1.657)	<0.001	1.327 (1.160-1.519)	<0.001
CLIP4	0.349 (0.278-0.438)	<0.001	0.357 (0.282-0.454)	<0.001	0.445 (0.363-0.546)	<0.001
DCTN1	0.491 (0.412-0.585)	<0.001	0.474 (0.396-0.568)	<0.001	0.482 (0.404-0.575)	<0.001
GAS2L2	1.593 (1.398-1.815)	<0.001	1.641 (1.435-1.876)	<0.001	1.524 (1.348-1.723)	<0.001
MACF1	0.722 (0.615-0.848)	<0.001	0.727 (0.613-0.861)	<0.001	0.833 (0.713-0.974)	0.022
MAPRE3	0.669 (0.543-0.823)	<0.001	0.636 (0.508-0.796)	<0.001	0.682 (0.567-0.820)	<0.001
NAV3	0.653 (0.571-0.747)	<0.001	0.663 (0.575-0.764)	<0.001	0.768 (0.680-0.867)	<0.001
SRCIN1	0.744 (0.671-0.824)	<0.001	0.723 (0.649-0.807)	<0.001	0.778 (0.710-0.852)	<0.001
TTBK1	0.522 (0.455-0.599)	<0.001	0.499 (0.430-0.579)	<0.001	0.542 (0.474-0.620)	<0.001
TTBK2	0.387 (0.323-0.465)	<0.001	0.385 (0.318-0.466)	<0.001	0.443 (0.374-0.525)	<0.001

B

Characteristics	HR (95% CI)KM-OS	P value	HR (95% CI)KM-DSS	P value	HR (95% CI)KM-PFI	P value
AMER2	0.280 (0.212-0.369)	<0.001	0.273 (0.204-0.366)	<0.001	0.384 (0.305-0.484)	<0.001
APC	0.392 (0.302-0.507)	<0.001	0.402 (0.306-0.529)	<0.001	0.495 (0.395-0.620)	<0.001
APC2	0.393 (0.303-0.509)	<0.001	0.404 (0.307-0.530)	<0.001	0.452 (0.361-0.567)	<0.001
CDK5RAP2	0.823 (0.643-1.054)	0.123	0.825 (0.635-1.072)	0.15	0.882 (0.708-1.098)	0.261
CEP104	1.348 (1.051-1.730)	0.019	1.375 (1.056-1.790)	0.018	1.273 (1.021-1.588)	0.032
CKAP5	0.719 (0.561-0.922)	0.009	0.738 (0.568-0.960)	0.024	0.797 (0.639-0.993)	0.043
CLASP1	0.529 (0.412-0.680)	<0.001	0.534 (0.409-0.697)	<0.001	0.586 (0.469-0.732)	<0.001
CLASP2	0.277 (0.211-0.364)	<0.001	0.256 (0.192-0.343)	<0.001	0.346 (0.275-0.437)	<0.001
CLIP1	1.124 (0.878-1.438)	0.354	1.130 (0.871-1.467)	0.357	1.196 (0.959-1.490)	0.112
CLIP2	0.911 (0.712-1.166)	0.46	0.898 (0.692-1.165)	0.417	0.969 (0.778-1.207)	0.779
CLIP3	0.367 (0.283-0.475)	<0.001	0.367 (0.279-0.483)	<0.001	0.521 (0.416-0.651)	<0.001
CTTNBP2	0.247 (0.187-0.324)	<0.001	0.237 (0.177-0.318)	<0.001	0.350 (0.278-0.441)	<0.001
DST	0.285 (0.217-0.374)	<0.001	0.275 (0.206-0.368)	<0.001	0.387 (0.308-0.488)	<0.001
FBXW11	0.433 (0.334-0.561)	<0.001	0.418 (0.317-0.550)	<0.001	0.516 (0.412-0.647)	<0.001
FILIP1	0.882 (0.689-1.130)	0.321	0.869 (0.668-1.129)	0.293	0.877 (0.704-1.093)	0.243
GAS2L1	0.670 (0.522-0.859)	0.002	0.699 (0.538-0.909)	0.008	0.748 (0.600-0.933)	0.01
KIF11	2.538 (1.943-3.316)	<0.001	2.589 (1.955-3.430)	<0.001	1.927 (1.538-2.416)	<0.001
KIF18A	5.865 (4.308-7.984)	<0.001	6.268 (4.504-8.724)	<0.001	3.755 (2.950-4.780)	<0.001
KIF18B	1.917 (1.485-2.476)	<0.001	1.911 (1.459-2.501)	<0.001	1.758 (1.404-2.201)	<0.001
KIF2C	5.306 (3.923-7.177)	<0.001	5.607 (4.070-7.723)	<0.001	3.437 (2.709-4.360)	<0.001
KNSTRN	2.318 (1.785-3.010)	<0.001	2.302 (1.749-3.029)	<0.001	1.766 (1.411-2.210)	<0.001
MAPRE1	1.259 (0.982-1.613)	0.069	1.275 (0.981-1.658)	0.069	1.155 (0.927-1.440)	0.198
MAPRE2	0.435 (0.337-0.562)	<0.001	0.409 (0.311-0.538)	<0.001	0.498 (0.397-0.623)	<0.001
NAV1	0.446 (0.345-0.577)	<0.001	0.435 (0.331-0.571)	<0.001	0.503 (0.401-0.630)	<0.001
NAV2	0.563 (0.438-0.724)	<0.001	0.578 (0.443-0.754)	<0.001	0.657 (0.527-0.820)	<0.001
NCKAP5	0.512 (0.399-0.658)	<0.001	0.507 (0.389-0.662)	<0.001	0.650 (0.522-0.811)	<0.001
NCKAP5L	0.926 (0.723-1.186)	0.543	0.913 (0.702-1.187)	0.496	0.760 (0.609-0.947)	0.015
PAFAH1B1	0.442 (0.342-0.570)	<0.001	0.437 (0.333-0.573)	<0.001	0.491 (0.392-0.615)	<0.001
PPP1R13L	1.425 (1.110-1.830)	0.005	1.444 (1.108-1.882)	0.007	1.338 (1.073-1.669)	0.01
PSRC1	5.803 (4.332-7.773)	<0.001	6.146 (4.494-8.406)	<0.001	4.374 (3.412-5.608)	<0.001
SLAIN1	0.617 (0.480-0.793)	<0.001	0.596 (0.456-0.778)	<0.001	0.681 (0.546-0.850)	<0.001
SLAIN2	2.162 (1.669-2.800)	<0.001	2.237 (1.701-2.943)	<0.001	1.866 (1.492-2.335)	<0.001
STIM1	0.935 (0.731-1.196)	0.591	0.951 (0.733-1.234)	0.705	0.879 (0.706-1.095)	0.25
SYBU	0.367 (0.282-0.478)	<0.001	0.360 (0.272-0.476)	<0.001	0.543 (0.434-0.680)	<0.001
TACC3	5.066 (3.765-6.817)	<0.001	5.196 (3.803-7.100)	<0.001	3.181 (2.509-4.033)	<0.001
TRIO	1.437 (1.121-1.843)	0.004	1.394 (1.073-1.813)	0.013	1.330 (1.067-1.658)	0.011
CLIP4	0.401 (0.309-0.521)	<0.001	0.389 (0.296-0.513)	<0.001	0.481 (0.383-0.602)	<0.001
DCTN1	0.446 (0.346-0.575)	<0.001	0.425 (0.324-0.557)	<0.001	0.519 (0.415-0.649)	<0.001
GAS2L2	1.465 (1.141-1.881)	0.003	1.456 (1.117-1.896)	0.005	1.303 (1.044-1.626)	0.019
MACF1	0.680 (0.531-0.871)	0.002	0.708 (0.545-0.920)	0.01	0.857 (0.688-1.068)	0.169
MAPRE3	0.762 (0.595-0.977)	0.032	0.758 (0.583-0.985)	0.038	0.750 (0.601-0.935)	0.011
NAV3	0.498 (0.385-0.642)	<0.001	0.498 (0.381-0.652)	<0.001	0.657 (0.526-0.820)	<0.001
SRCIN1	0.555 (0.431-0.715)	<0.001	0.515 (0.393-0.675)	<0.001	0.597 (0.477-0.747)	<0.001
TTBK1	0.487 (0.377-0.628)	<0.001	0.457 (0.348-0.599)	<0.001	0.568 (0.455-0.710)	<0.001
TTBK2	0.344 (0.264-0.450)	<0.001	0.341 (0.257-0.452)	<0.001	0.453 (0.361-0.569)	<0.001

Figure S2. Identification of prognostic-related DEMPERGs in glioma. Related to Figure 6A. (**A**,**B**) Forest plots of univariate Cox (A) and KM analyses (B) of DEMPEAGs with OS, DSS, and PFI. Yellow and blue words represented upregulated and downregulated genes in normal and glioma tissues, and yellow and blue dots represented risky and protective genes in glioma, respectively.

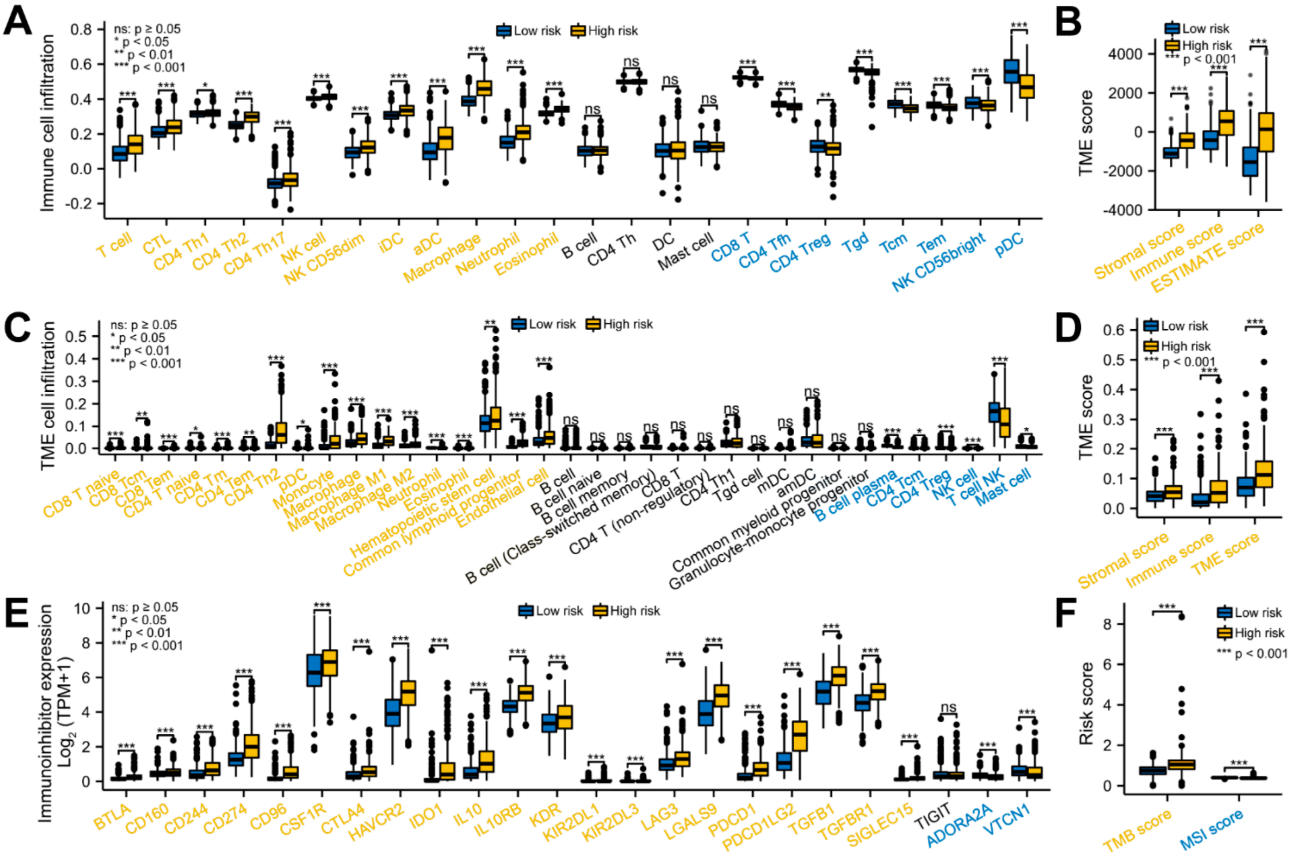

Figure S3. Different TME infiltrations between the two risk groups in glioma. Related to Figure 8. (**A**,**B**) Histograms of immune cell infiltration (A) and TME scores (B) using ssGSEA and ESTIMATE algorithm between the two risk groups. (**C**,**D**) Histograms of TME cell infiltration (C) and TME scores (D) using the xCell algorithm between the two risk groups. (**E**,**F**) Histograms of immunoinhibitor expression (E), TMB and MSI score (F) between the two risk groups.
